# Invariant Characteristics of Carcinogenesis

**DOI:** 10.1371/journal.pone.0140405

**Published:** 2015-10-14

**Authors:** Simon Sherman, Nirosha Rathnayake, Tengiz Mdzinarishvili

**Affiliations:** 1 Eppley Institute for Research in Cancer, University of Nebraska Medical Center, Omaha, Nebraska, United States of America; 2 College of Public Health, University of Nebraska Medical Center, Omaha, Nebraska, United States of America; 3 School of Natural Sciences and Engineering, Ilia State University, Tbilisi, Georgia; University of Torino, ITALY

## Abstract

Carcinogenic modeling is aimed at mathematical descriptions of cancer development in aging. In this work, we assumed that a small fraction of individuals in the population is susceptible to cancer, while the rest of the population is resistant to cancer. For individuals susceptible to cancer we adopted methods of conditional survival analyses. We performed computational experiments using data on pancreatic, stomach, gallbladder, colon and rectum, liver, and esophagus cancers from the gastrointestinal system collected for men and women in the SEER registries during 1975–2009. In these experiments, we estimated the time period effects, the birth cohort effects, the age effects and the population (unconditional) cancer hazard rates. We also estimated the individual cancer presentation rates and the individual cancer resistance rates, which are, correspondingly, the hazard and survival rates conditioned on the susceptibility to cancer. The performed experiments showed that for men and women, patterns of the age effects, the individual cancer presentation rates and the individual cancer resistance rates are: (i) intrinsic for each cancer subtype, (ii) invariant to the place of living of the individuals diagnosed with cancer, and (iii) well adjusted for the modifiable variables averaged at a given time period. Such specificity and invariability of the age effects, the individual cancer presentation rates and the individual cancer resistance rates suggest that these carcinogenic characteristics can be useful for predictive carcinogenic studies by methods of inferential statistics and for the development of novel strategies for cancer prevention.

## Introduction

Throughout the more than half century of the carcinogenic modeling history, a large number of different models have been proposed (see, for instance, [1–11]). As a rule, these models utilize the “mutation-centric” paradigm, postulating that all individuals in the population are susceptible to cancer and that cancer occurs in those individuals who, by chance, got a “bad set” of mutations. Meanwhile, the existing models are challenged by a decline of the age-specific incidence rates in very old ages [12], and some models [6–11] assume that people have different susceptibility to cancer due to an unobserved random factor. To account for this factor and to better fit the observed data, a non-negative random variable (a frailty) is implemented in those models. However, this requires a determination of parameters presenting the distribution of frailty. To improve fitting, researchers also add new biological details into the conjectured mechanisms of carcinogenesis [6, 7] that make the modeling a complicated, computationally unstable problem with poorly identifiable parameters [11]. Overall, the carcinogenic models developed so far are useful for descriptive analyses, but their effectiveness for the inferential studies is limited.

To improve the performance of the carcinogenic modeling, the main concepts of carcinogenic modeling can be enriched by adapting the basic concepts of survival analysis (such as hazard rates, survival rates, and probability density rates) [13,14]. In fact, carcinogenic modeling and cancer survival modeling have common mathematical roots. Both are aimed at analyzing the hazards of the experience of distinct events during the corresponding waiting time. In carcinogenic modeling, the event is a diagnosis of cancer (presentation of cancer confirmed by clinical means) in an individual and the time to event (waiting time) is the age at which this individual was diagnosed with the cancer. In cancer survival modeling, the event is the death of a cancer patient and the time to the event is the number of months passed from the diagnosis of cancer until the patient’s death.

Despite a noticeable similarity between the mathematical concepts of carcinogenic modeling and cancer survival modeling, a mathematical formalism used in survival analysis cannot be directly adapted for carcinogenic modeling. In conventional survival analysis, all cancer patients experience the event (death), while in carcinogenic modeling the vast majority of individuals of the considered population do not experience the cancer presentation. This challenge was overcome in [13,14] by postulating a dichotomous susceptibility to cancer in the population. According to that postulate, only a small fraction of individuals can experience cancer during their lifetime, while the majority of the population escapes cancer. It should be noted that the idea of the dichotomous susceptibility to cancer in the population was initially proposed and then immediately rejected nearly 45 years ago in [15]. However, a recent study performed in [14], showed that the rejection was erroneous.

In the present work, we further implemented a mathematical formalism of survival analysis into the carcinogenic modeling. To do this, we analyzed how the conditional hazard rates (individual cancer presentation rates) and the conditional survival rates (individual cancer resistance rates) of individuals susceptible to cancer depend on cancer site, sex, the time period of cancer diagnostics and the geographic area of living. For this purpose, we performed two series of computational experiments on six cancers from the gastrointestinal (GI) system. In these experiments, data on pancreatic, stomach, gallbladder, colon and rectum, liver and esophagus cancers were used as test beds. The experiments were performed using data collected in the Surveillance, Epidemiology, and End Results (SEER) databases [16] for men and women diagnosed with the considered cancers during 1975–2009 in different geographic areas of the U.S.A. For simplicity, we considered sex and race as unmodifiable variables, while other variables (such as geographic area of living, time period of cancer diagnosis and other factors) that can influence on cancer presentation–as modifiable (environmental) variables.

The results of these computational experiments showed that for men and women the individual cancer presentation rates and the individual cancer resistance rates are intrinsic for each cancer site and are nearly independent of the time period of cancer diagnosis and the geographic area of cancer presentation. This suggests that these rates are independent of environmental variables. If so, these rates are analogues of the baseline hazard rates and the baseline survival rates in conventional survival analysis. The latest points on that the individual cancer presentation rates and the individual cancer resistance rates can be useful for predictive studies of carcinogenesis by methods of inferential statistics and for the development of novel strategies for cancer prevention.

## Materials and Methods

### Population and individual hazard rates

The age-specific incidence rates (crude rates) are used for analyzing cancer occurrence in aging. These rates are characterized by a number of new cases with a distinct type of cancer diagnosed during a specified time period within the age-specific population (the population of individuals equally distributed in specified age intervals). Due to the rareness of cancer occurrence, the age-specific incidence rates are often adjusted to 100,000 person-years. In practice, the age-specific incidence rates are determined as a ratio of the observed cancer cases in the specified five-year time period, divided by the total person-years at risk, in the subpopulations of individuals, the ages of which belong to the sequential five-year age intervals. For the SEER databases, the five-year age intervals in which the number of cases exceeds 15 are traditionally used. Usually, for adult cancers, such age intervals start at the age of 20 and end at the age of 99.

To increase a statistical power, the age-specific incidence rates of cancers are collected during a long time period that contains many five-year time periods. To determine the age-specific hazard rates, the age-specific incidence rates, collected during those time periods, are corrected on the age-period-cohort (APC) effects [2–4,17,18]. The obtained hazard rates are referred to as the population hazard rate [13,14]. Analogously, for individuals susceptible to cancer, the age-specific cancer hazard rates are referred to as the individual hazard rates [13,14], which are conditional (conditional to susceptibility) rates. In the present work, to determine the APC effects as well as the population and individual hazard rates (individual cancer presentation rates), the web tool developed in [19], *CancerHazard@Age*, was used.

### Adapting a mathematical formalism of survival analysis for carcinogenic modeling

In this work, we used the concepts and designations of the survival analysis that were adapted in [13,14] for purposes of carcinogenic modeling. By *S*(*t*) we denoted a conditional survival function that an individual "survives" from getting a particular type of cancer at the age *t*, given that this individual belongs to the pool of individuals susceptible to cancer, and we called *S*(*t*) the individual survival function. Analogously, we denoted by *h*(*t*) and *f*(*t*) the conditional hazard function and the conditional probability density function, correspondingly. We also called *h*(*t*) the individual hazard function (as well as the individual cancer presentation function) and *f*(*t*) - the probability density function of individual cancer presentation. According to the conventional survival analysis formalism, *S*(*t*), *h*(*t*) and *f*(*t*) are related in the following way [20]:

h(t)=f(t)/S(t) (1)

f(t)=−dS(t)/dt (2)

$$S\left(t\right)=\text{exp}\left(-\int_{0}^{t}h(z)dz\right)=\text{exp}[-H(t)],$$ (3)

where

$$H(t)=\int_{0}^{t}\;h(z)dz$$ (4)

is the cumulative individual hazard function.

By *S*_*U*_ (*t*) we denoted an unconditional survival (cancer resistance) function showing that an individual, randomly chosen from the population, did not experience the event (cancer presentation) at the age *t* (i.e. this individual did not develop cancer up to that age). We called *S*_*U*_ (*t*) the population (unconditional) cancer resistance function. Analogously, we denoted the unconditional cancer hazard function (or population cancer hazard function) by *h*_*U*_ (*t*). According to [20], *S*_*U*_ (*t*) and *S*(*t*) are related as follows:

$$S_{U}\left(t\right)=\left(1-p\right)\cdot 1+p\cdot S\left(t\right)=1-p+pS\left(t\right)$$ (5)

and

$$S\left(t\right)=\left(1/p\right)\left[S_{U}\left(t\right)+p-1\right],$$ (6)

where *p* is the probability that a randomly chosen individual is susceptible to cancer and 1 − *p* is the probability that this individual is resistant to cancer. Note, *p* can also be considered as the relative size of the pool of the individuals susceptible to cancer [14].

The unconditional cancer hazard function, *h*_*U*_ (*t*), showing that an individual, randomly chosen from the whole population, gets cancer at the age *t* can be presented in the following way [14]:

$$h_{U}\left(t\right)=\left[-dS_{U}\left(t\right)/dt\right]/S_{U}\left(t\right)=-d\;\text{ln}\left[S_{U}\left(t\right)\right]/dt=pf\left(t\right)/\left[1-p+pS\left(t\right)]=ph\left(t\right)\text{exp}[-H\left(t\right)\right]/\left\{1-p+p\text{exp}\left[-H\left(t\right)\right]\right\}=ph\left(t\right)/\left\{p+\left(1-p\right)\;\text{exp}\left[H\left(t\right)\right]\right\}$$ (7)

The conditional cancer hazard function, *h*(*t*), which shows that an individual, randomly chosen from the pool of individuals susceptible to cancer, is diagnosed with cancer at the age *t* is presented as [14]:

$$h\left(t\right)=\left[-dS\left(t\right)/dt\right]/S\left(t\right)=h_{U}\left(t\right)S_{U}\left(t\right)/\left[S_{U}\left(t\right)+p-1\right]=h_{U}\left(t\right)\;\text{exp}\left[-H_{U}\left(t\right)\right]/\left\{\text{exp}\left[-H_{U}\left(t\right)\right]+p-1\right\}=h_{U}\left(t\right)/\left\{1+\left(p-1\right)\;\text{exp}\left[H_{U}\left(t\right)\right]\right\}$$ (8)

where:

$$H_{U}(t)=\int_{0}^{t}\;h_{U}(z)dz$$ (9)

is the cumulative unconditional cancer hazard function.

When *p* (the relative size of the pool of individuals susceptible to cancer) is small, the overall cumulative cancer hazard, *H*_*UO*_, can be presented (with a first-order approximation) as [14]:

$$H_{UO}=\int_{0}^{\infty }\;h_{U}(t)dt=-\text{ln}(1-p)=p.$$ (10)

Also, for small *p*, *h*_*U*_ (*t*) and *h*(*t*) are related in the following way [14]:

$$h_{U}\left(t\right)=pf\left(t\right)/\left[1-p+pS\left(t\right)\right]=pf\left(t\right)=ph\left(t\right)\;\text{exp}\left[-H\left(t\right)\right]$$ (11)

$$h\left(t\right)=h_{U}\left(t\right)/\left\{1+\left(p-1\right)\;\text{exp}\left[H_{U}\left(t\right)\right]\right\}=h_{U}\left(t\right)/\left\{1+\left(p-1\right)\left[1+H_{U}\left(t\right)\right]\right\}=h_{U}\left(t\right)/\left\{1+\left(H_{UO}-1\right)\left[1+H_{U}\left(t\right)\right]\right\}=h_{U}\left(t\right)/\left[H_{UO}-H_{U}\left(t\right)\right].$$ (12)

From (12) it follows that an empirical estimate (denoted by sign “^”) of the conditional cancer hazard function, $\hat{h}\left(t\right)$, can be obtained by the following formula:

$$\hat{h}(t)={\hat{h}}_{U}(t)/[{\hat{H}}_{UO}-{\hat{H}}_{U}(t)].$$ (13)

The standard errors of $\hat{h}(t)$, $SE[\hat{h}(t)]$, can be determined as [14]:

$$S{\hat{E}}^{2}\left[\hat{h}\left(t\right)\right]=\left\{{\hat{h}}_{U}^{2}\left(t\right)/{\left[{\hat{H}}_{UO}-{\hat{H}}_{U}\left(t\right)\right]}^{2}\right\}\left\{S{\hat{E}}^{2}\left[{\hat{h}}_{U}\left(t\right)\right]/{\hat{h}}_{U}^{2}\left(t\right)+S{\hat{E}}^{2}\left[{\hat{H}}_{UO}-{\hat{H}}_{U}\left(t\right)\right]/{\left[{\hat{H}}_{UO}-{\hat{H}}_{U}\left(t\right)\right]}^{2}\right\}.$$ (14)

In this work, we use both functions and rates: for functions, *t* (age) is a continuous variable, while for rates, *t*_*i*_ is a discrete variable presenting the corresponding *n* successive age intervals indexed as *i* = 1,2, …, *n*. Also, we called the conditional (individual) cancer hazard rates as the individual cancer presentation rates and the conditional cancer resistance rates as the individual cancer resistance rates to denote that we deal with the problem of cancer occurrence (but not with the problem of cancer survival).

### Data preparation

In this work, we used the SEER databases [16] containing information on cancer cases collected 1975–2009 in the U.S.A. Data gathered in the following nine geographical areas were used in our study: San Francisco-Oakland SMSA, Connecticut, Detroit (Metropolitan), Hawaii, Iowa, New Mexico, Seattle (Puget Sound), Utah, and Atlanta (Metropolitan). Below, the "Entire" region refers to these nine geographic areas, the “Eastern” region refers to four areas (Atlanta, Connecticut, Detroit and Iowa), while the “Western” region refers to the other five areas (San Francisco-Oakland SMSA, Seattle, Hawaii, New Mexico, and Utah). Data on patients diagnosed with only the first, primary, microscopically-confirmed cancers were considered.

For extraction of data and for primary data processing, the statistical software package, SEER*Stat version 8.1.5 [21] was used. With this software, the cases stratified by the distinct cancer sites (pancreatic, stomach, gallbladder, colon and rectum, liver and esophagus), gender (men and women) and geographic region (Entire, Eastern and Western regions) were determined and combined in seven (*j* = 1, …, 7), five-year (cross-sectional) time-period intervals (specified as: 1975–1979; 1980–1984; … 2005–2009). In this work, only the cases diagnosed at ages from 20 to 99 were used. Finally, the chosen cases were fractioned into *n* = 16 groups, corresponding to the five-year age intervals, Δ = 5 years, ranging from 20 to 99 (*i* = 1, …, *n* and *n* = 16).

For each of the considered cancer sites (pancreatic, stomach, gallbladder, colon and rectum, liver and esophagus), six case matrices (*Cases*) of 16x7 sizes (three matrices with numbers of men and three matrices with numbers of women who have been diagnosed within each of the 16 age intervals during each of the seven specified time-period intervals with the given type of cancer in the Entire, Eastern and Western regions, correspondingly) were obtained. (A detailed description on how to prepare the case matrices is given in [19].) The population distributions of men and women and the distributions of the number of the GI cancer cases diagnosed during 1975–2009 within men and women, who have lived in Eastern and Western regions, are given in S1 Appendix. For the Entire region, the population distributions and the distributions of the GI cancer cases diagnosed during 1975–2009 within men and women were obtained by a simple summation of the corresponding distributions in the Eastern and Western regions. For example, Table 1 shows the number of occurrences of stomach cancer in the five-year long age intervals within the 20–99 old men, who have lived in the Entire region during 1975–2009, while Table 2 shows the distribution of the men population in the Entire region during that time.

**Table 1:** Distribution of the number of occurrences (*O*_*i*,*j*_) of stomach cancer in men living in the Entire region during seven time periods of 1975–2009.

Age		Number of cancers in the time periods (*j* = 1,…7)						
Index, *i*	Interval	1975–79	1980–84	1985–89	1990–94	1995–99	2000–04	2005–09
1	20–24	3	6	7	10	6	7	4
2	25–29	18	26	3	16	19	24	21
3	30–34	23	28	49	42	40	38	47
4	35–39	63	61	64	84	90	85	91
5	40–44	118	102	139	162	158	137	142
6	45–49	231	200	209	210	240	289	314
7	50–54	410	361	312	303	324	392	442
8	55–59	576	573	522	435	443	504	558
9	60–64	795	783	780	651	569	555	633
10	65–69	798	940	866	843	710	649	641
11	70–74	742	830	927	950	825	696	633
12	75–79	649	644	759	711	780	684	631
13	80–84	447	491	469	471	503	581	498
14	85–89	206	253	239	276	260	277	308
15	90–94	71	76	82	94	85	78	94
16	95–99	12	22	17	17	20	12	11

**Table 2:** Distribution of the male populations (*Pop*_*i*,*j*_) living in the Entire region during seven time periods of 1975–2009.

Age		Populations in the time periods (*j* = 1,…7)						
Index, *i*	Interval	1975–79	1980–84	1985–89	1990–94	1995–99	2000–04	2005–09
1	20–24	4823448	5066913	4740361	4468579	4345198	4794672	4950981
2	25–29	4557117	5107569	5314620	5037570	4919617	4721690	4971495
3	30–34	3854083	4647582	5174288	5519031	5373101	5151259	4785983
4	35–39	3031712	3764909	4578026	5224678	5602105	5283839	5053627
5	40–44	2655844	2980012	3763370	4646871	5199589	5447194	5177116
6	45–49	2688338	2573983	2944425	3692897	4529835	5046921	5308329
7	50–54	2757647	2596258	2495095	2851472	3635290	4432905	4911526
8	55–59	2517854	2563881	2413126	2360030	2719212	3433799	4234026
9	60–64	2078337	2251967	2289385	2217312	2184372	2488027	3190864
10	65–69	1595444	1788748	1963592	2014472	1958011	1927699	2259769
11	70–74	1134899	1291607	1445855	1621767	1694793	1674177	1690403
12	75–79	736030	843325	982036	1136567	1302277	1380812	1375331
13	80–84	440318	469740	542595	650905	786468	920646	1010045
14	85–89	202239	229444	252456	294440	362880	435645	539711
15	90–94	69129	78429	86295	100646	124040	148913	184484
16	95–99	15213	17260	18991	22149	27297	32771	40599

Further, using the databases introduced in [22,23], six population matrices (*Populations*) of 16x7 sizes (three matrices with numbers of men and three matrices with numbers of women in ages within each of the 16 age intervals who have lived during each of the seven considered time-period intervals in the Entire, Eastern and Western regions, correspondingly) were created. The procedures for determining the population matrices are described in detail in [19].

### Data processing

The *Cases* and *Populations* matrices, saved as the tab-separated-value files, were used as input data for the *CancerHazard@Age* web tool [19] that is freely available at http://registry.unmc.edu/CHA/. The *CancerHazard@Age* was used while performing several computational experiments, the outcomes of which are described in the Results and Discussion section. To work with the *CancerHazard@Age*, the values of the following variables were input: *Title* (title of the computational experiment); *Start Age* (the youngest age, of the first age interval, which was taken equal to 20); *Start Year* (the first year, of the first time period interval, which was taken equal to 1975); and *Time Interval* (the width, Δ, of the time period intervals, which was taken equal to 5). In our computational experiments, the *CancerHazard@Age* was used in a regime of the manual anchoring. To do this, the values of two additional variables were input: *Period Index* (*j*, the index of the anchored time period) and *Age Index* (*i*, the index of the anchored age interval). In our computational experiments (see below), we used the values of the *Period Index* equal to four (when the 1990–1994 time-period was used as anchors) or equal to seven (when the 2005–2009 time-period was used as an anchor). The value of the *Age Index* was taken equal to 11 (i.e. the age interval of 70–74 was used as an anchor). Note, the index for the anchored birth-cohort is determined by the *CancerHazard@Age* as *k* = *j* − *i* + *n* [19]. For instance, in this work we used *j* = 4 (as well as *j* = 7), *i* = 11 and *n* = 16. For *j* = 4, *i* = 11 and *n* = 16, the index *k* of the anchored birth-cohort is equal to nine, while for *j* = 7, *i* = 11 and *n* = 16, the value of *k* is equal to 12.

## Results and Discussion

### Description of computational experiments

We performed two series of computational experiments. The goal of the first series of experiments was to analyze how the time-period effects, the birth-cohort effects, the age-at-diagnosis effects, the population cancer hazard rates, the individual cancer hazard rates (individual cancer presentation rates), and the individual cancer survival rates (individual cancer resistance rates) for the pancreatic, stomach, gallbladder, colon and rectum, liver, and esophagus cancers depend upon the time period (1990–1994 or 2005–2009) at which these cancers were diagnosed. The goal of the second series of experiments was to analyze how the aforementioned carcinogenic characteristics of the considered GI cancers depend upon the geographic areas (Eastern and Western) in which these cancers were diagnosed.

The first series is comprised of two sets of computational experiments. In each of those sets, the 16x7 matrixes of *Cases* (presenting distributions of numbers of each of the considered GI cancers diagnosed in men and women in 16 distinct five-year age intervals during seven time periods from 1975 to 2009) and *Populations* of men and women living in the nine geographic areas, called the Entire region, were used as input data for the *CancerHazard@Age* web tool [19]. As an example, Table 1 presents the number of occurrences of stomach cancer in men living in the Entire region during 1975–2009 and Table 2 shows the distribution of all men living in that region during 1975–2009.

In the first set of computational experiments, the time-period effects, the birth-cohort effects, the age-at-diagnosis effects, the population cancer hazard rates, and the individual cancer presentation rates for the pancreatic, stomach, gallbladder, colon and rectum, liver, and esophagus cancers, anchored to the 1990–1994 time period were determined. To do this (as described in the Materials and Methods section), the value of the *Period Index* equal to four was used as the input parameter for the CancerHazard@Age. In the second set of computational experiments, the aforementioned carcinogenic characteristics of the considered GI cancers, anchored to the 2005–2009 time period, were determined. To do this, the value of the *Period Index* equal to seven was used as the input parameter for the CancerHazard@Age. Note, for these two time periods, the resistance (survival) rates and their standard errors were estimated by the equations (41) and (42), presented in [13].

The second series is also comprised of two sets of computational experiments. In the first set of that series, the 16x7 matrices of *Cases* (presenting distributions of numbers of cases for each of the considered cancers diagnosed in 16 distinct five-year age intervals during seven time periods from 1975 to 2009) and *Populations* (men and women) living in the nine geographic areas, the matrices of *Cases* and *Populations* for the Eastern geographic area were used as input data for the CancerHazard@Age, while in the second set experiments, the matrices of *Cases* and *Populations* for the Western geographic area were used. In both sets of experiments, the time-period effects, the birth-cohort effects, the age-at-diagnosis effects, the population cancer hazard rates, the individual cancer presentation rates and the individual cancer resistance rates for the pancreatic, stomach, gallbladder, colon and rectum, liver and esophagus cancers, anchored to the 1990–1994 time period were determined. To do this, the value of the *Period Index* equal to four was used as the input parameter for the CancerHazard@Age tool.

### Dependency of the carcinogenic characteristics of the GI cancers upon the time-period at diagnosis

**Fig 1:**
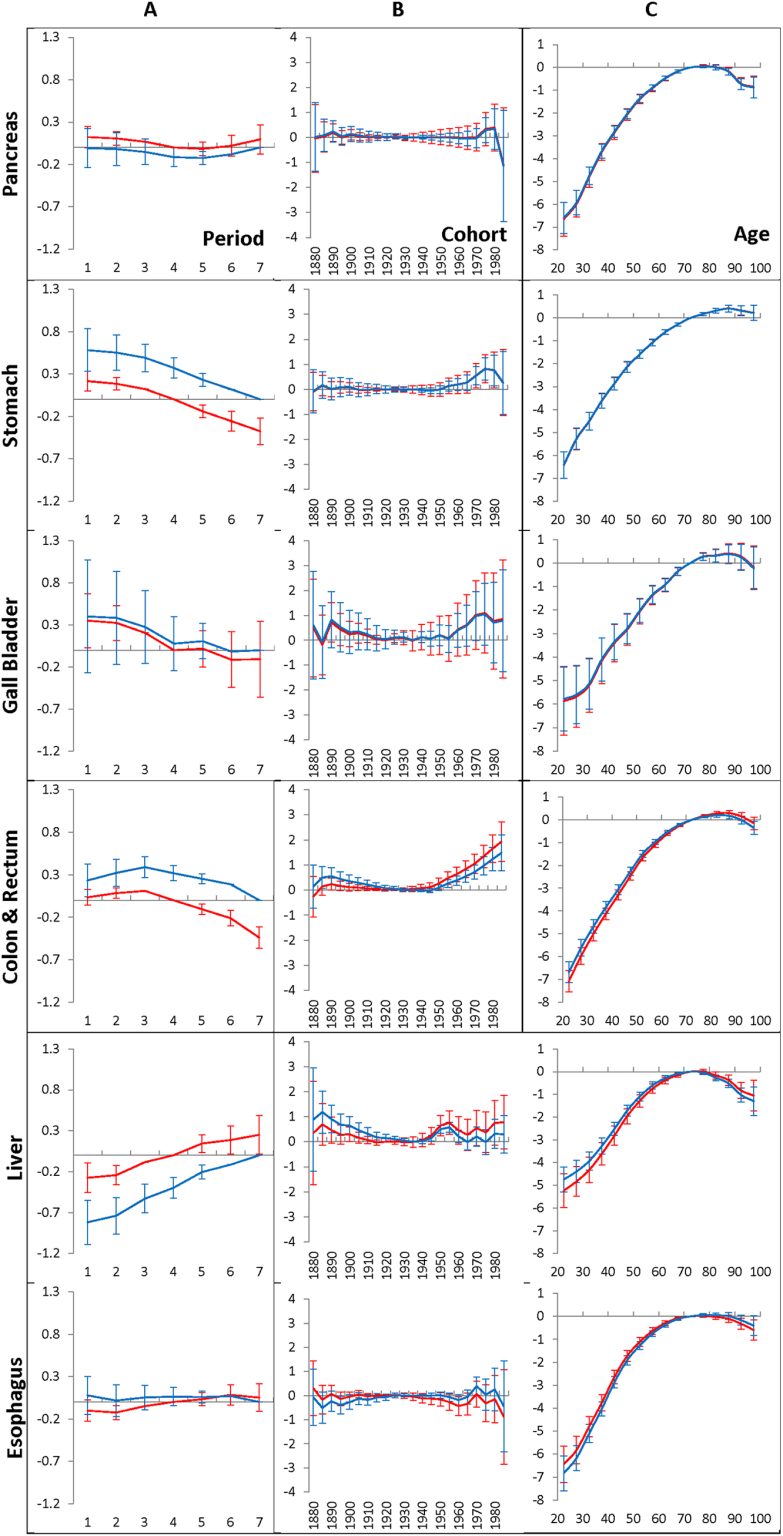
Time-period effects (A panel), birth-cohort effects (B panel) and age-at-diagnosis effects (C panel) for men who have lived in the Entire region and diagnosed with GI cancers during 1975–2009.

**Fig 2:**
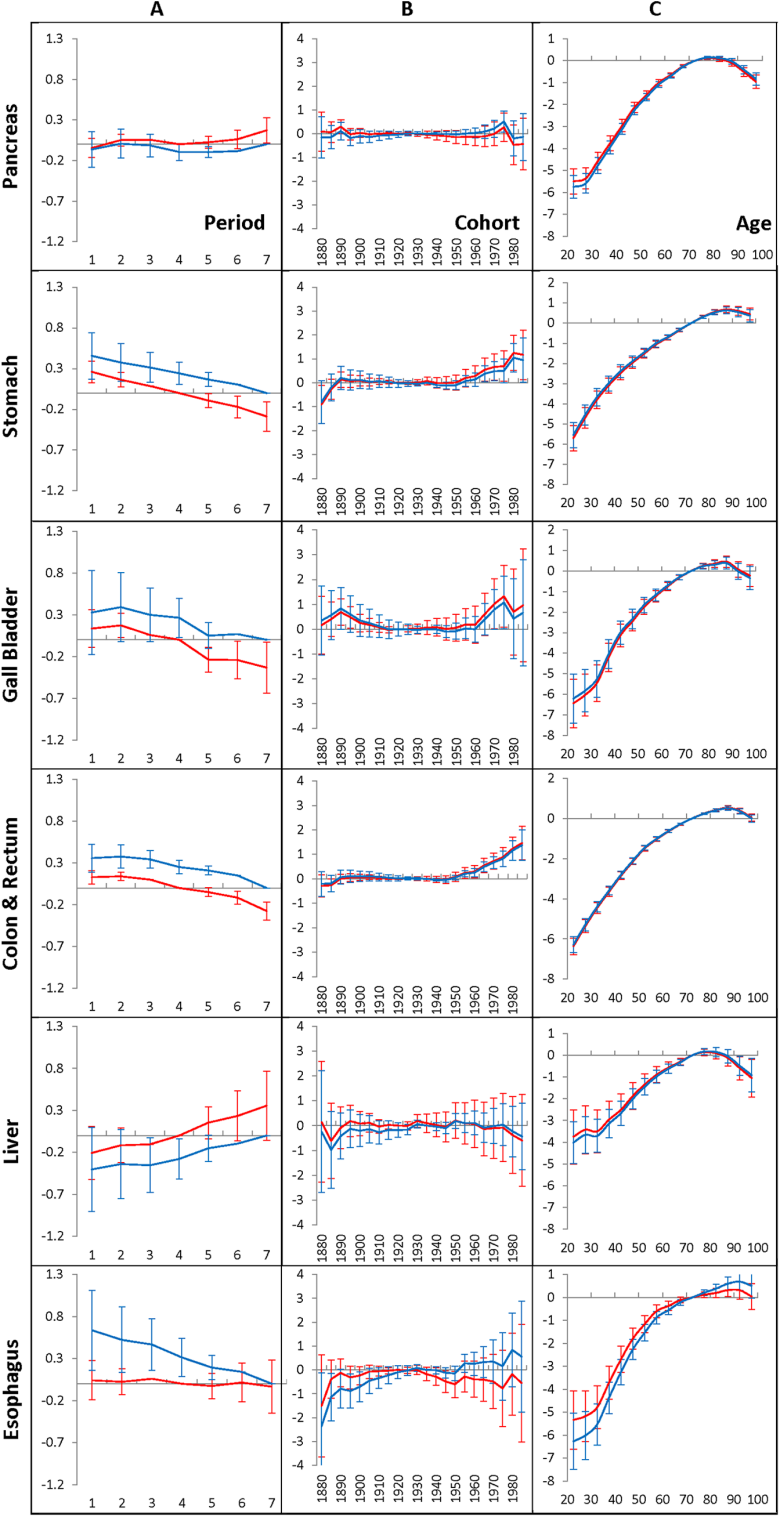
Time-period effects (A panel), birth-cohort effects (B panel) and age-at-diagnosis effects (C panel) for women who have lived in the Entire region and diagnosed with GI cancers during 1975–2009.

**Fig 3:**
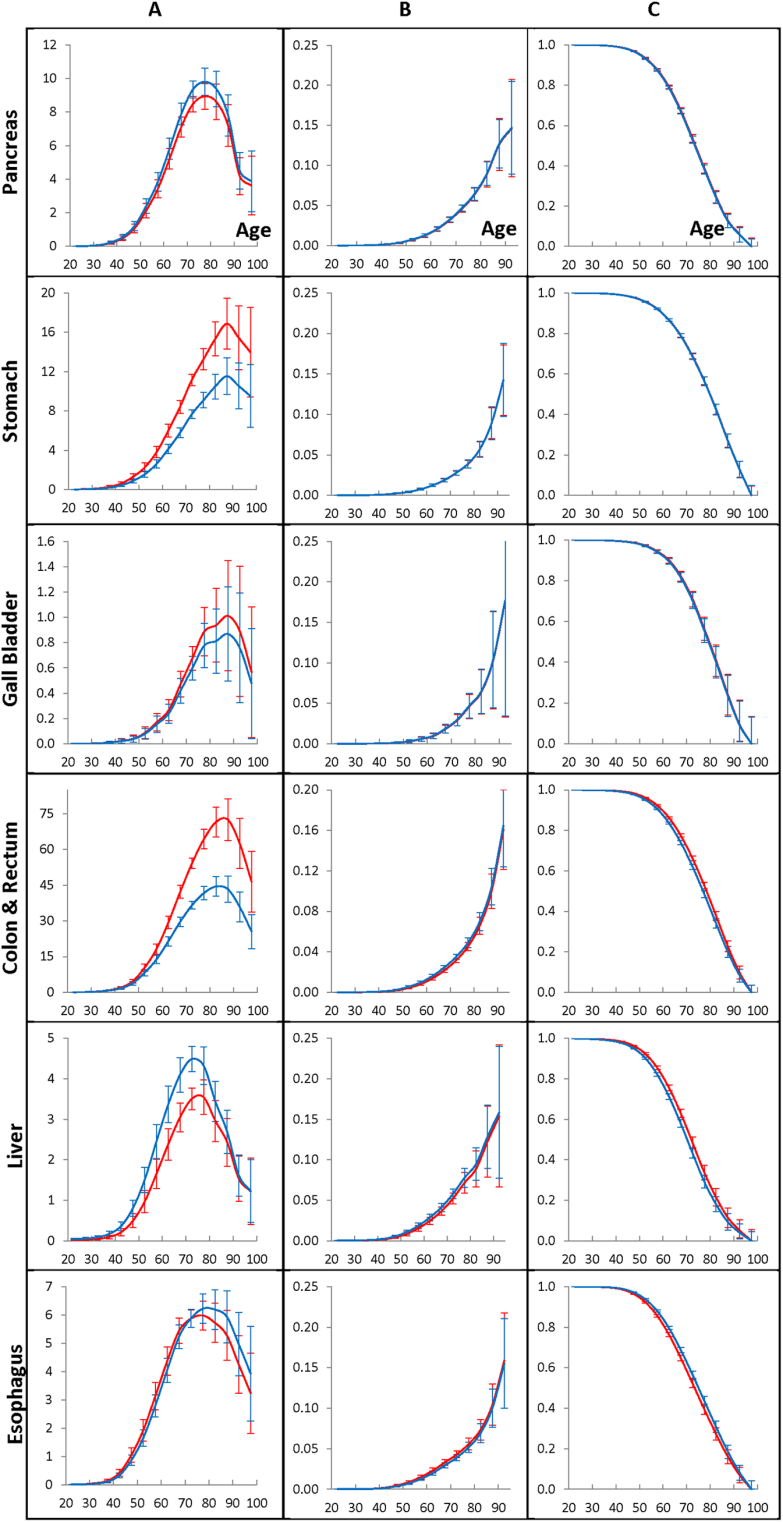
Population cancer hazard rates (A panel), individual cancer presentation rates (B panel), and individual cancer resistance rates (C panel) for men who have lived in the Entire region and diagnosed with GI cancers during 1975–2009.

**Fig 4:**
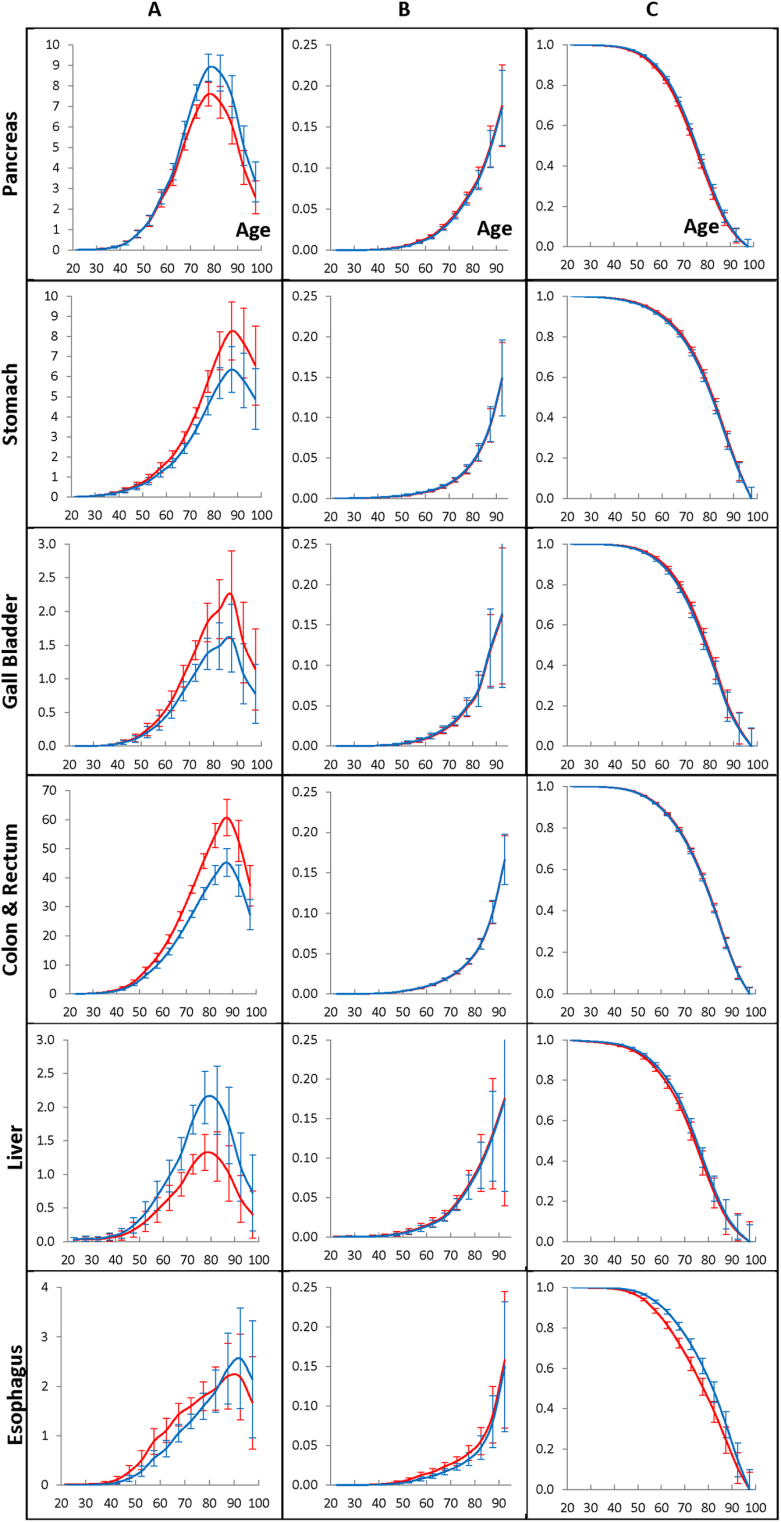
Population cancer hazard rates (A panel), individual cancer presentation rates (B panel), and individual cancer resistance rates (C panel) for women who have lived in the Entire region and diagnosed with GI cancers during 1975–2009.

Outcomes from the first series of computational experiments (see the Materials and Methods section) are exhibited in Fig 1, Fig 2, Fig 3 and Fig 4. Fig 1 shows the time-period effects (panels A), the birth-cohort effects (panels B), and the age-at-diagnosis effects (panels C) on the hazard of occurrence of pancreatic, stomach, gallbladder, colon and rectum, liver and esophagus cancers diagnosed in men who have lived during 1975–2009 in the Entire region, which includes the San Francisco-Oakland SMSA, Connecticut, Detroit, Hawaii, Iowa, New Mexico, Seattle, Utah, and Atlanta geographic areas. The effects are estimated by the time period of 1990–1994 (red lines) or the time period of 2005–2009 (blue lines), as well as the age interval of 70–74 taken as anchors. The error bars indicate 95% of the confidence intervals of the corresponding effects. The time-period effects, the birth-cohort effects and age-at-diagnosis effects that relate to the corresponding anchors, as well as the time period effects foregoing the anchored time period are presented without error bars (i.e. they are taken equal to zero). Fig 2 shows the similar effects in women.

Fig 3 exhibits the population cancer hazard rates (A panels), the individual cancer presentation rates (B panels), and the individual cancer resistance rates (C panels) of the pancreatic, stomach, gallbladder, colon and rectum, liver and esophagus cancers diagnosed in men who have lived during 1975–2009 in the Entire region. The rates are estimated by the time period of 1990–1994 (red lines) or the time period of 2005–2009 (blue lines), as well as the age interval of 70–74 taken as anchors. The error bars indicate 95% of the confidence intervals of the corresponding rates. Fig 4 shows the similar effects in women.

As can be seen from Fig 1 and Fig 2, for each of the considered GI cancers diagnosed in men and women during 1975–2009, the time-period effects (A panels) and the birth-cohort effects (B panels) are small. This phenomenon takes place independent of which time period (1990–1994 or 2005–2009) is used as an anchor. The birth-cohort effects for pancreatic, stomach, gallbladder, liver, and esophagus cancers are negligible: this is evidenced by the fact that the corresponding error bars contain the value of zero. For the colon and rectum cancers, most of the birth-cohort effects are also negligible. However, error bars of several effects of the youngest cohorts are small but significant. This is evidenced by the fact that the corresponding error bars do not include the value of zero of these effects.

In contrast to the time-period effects and to the birth-cohort effects, the age effects strongly influence the occurrence of each of the considered GI cancers in men, as well in women (see the C panels in Fig 1 and Fig 2, correspondingly). This influence increases considerably with age up to 70, evens out at the ages of 70–85 and slightly falls at very old ages. The other distinguishable feature of the age effects is that their patterns are unique for each considered cancer site and nearly independent of the time periods used for anchoring.

For the considered cancers, the population cancer hazard rates anchored to the 1990–1994 and 2005–2009 time periods are shown in the A panels of Fig 3 (for men) and Fig 4 (for women). As can be seen from these figures, the population cancer hazard rates of the pancreatic, stomach, gallbladder, colon and rectum, liver and esophagus cancers have similar shapes. These rates are gently increasing up to the ages of 40–50 and then become fast growing up to the ages of 65–70. At the ages of 70–85, the population cancer hazard rates flatten out, reach their maximum and then fall at the very old ages. The locations of these maximum amounts depend on the cancer site and gender. The values of the population cancer hazard rates depend on the time-period of cancer diagnosis used as the anchor and are almost proportionally changing in all of the five-year age intervals. For men and women susceptible to cancer, the individual cancer presentation rates are exponentially growing with age (see the B panels of Fig 3 and Fig 4, correspondingly), while for the individuals susceptible to cancer, the individual cancer resistance rates are continuously decreasing with age (see the C panels of Fig 3 and Fig 4, correspondingly).

### Dependency of the carcinogenic characteristics of the GI cancers upon the geographic area

**Fig 5:**
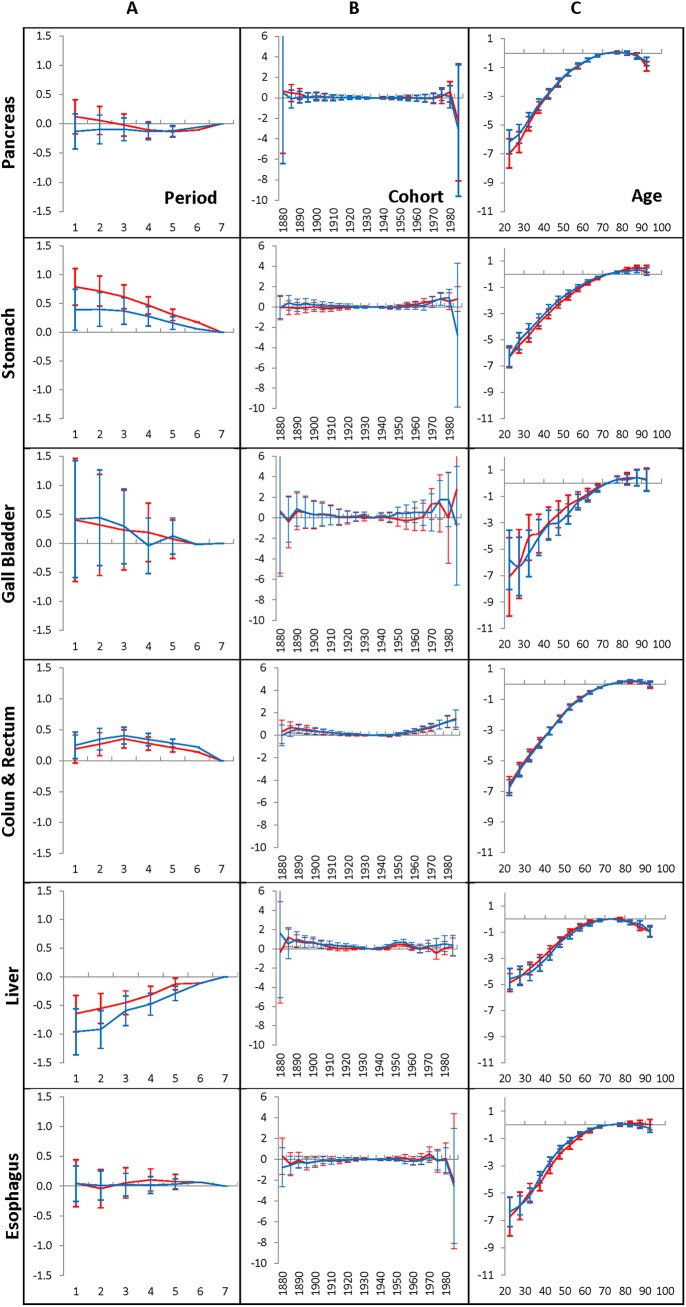
Time-period effects (A panel), birth-cohort effects (B panel) and age-at-diagnosis effects (C panel) for men who have lived in the Eastern (blue lines) and Western (red lines) geographic regions and diagnosed with GI cancers during 1975–2009.

**Fig 6:**
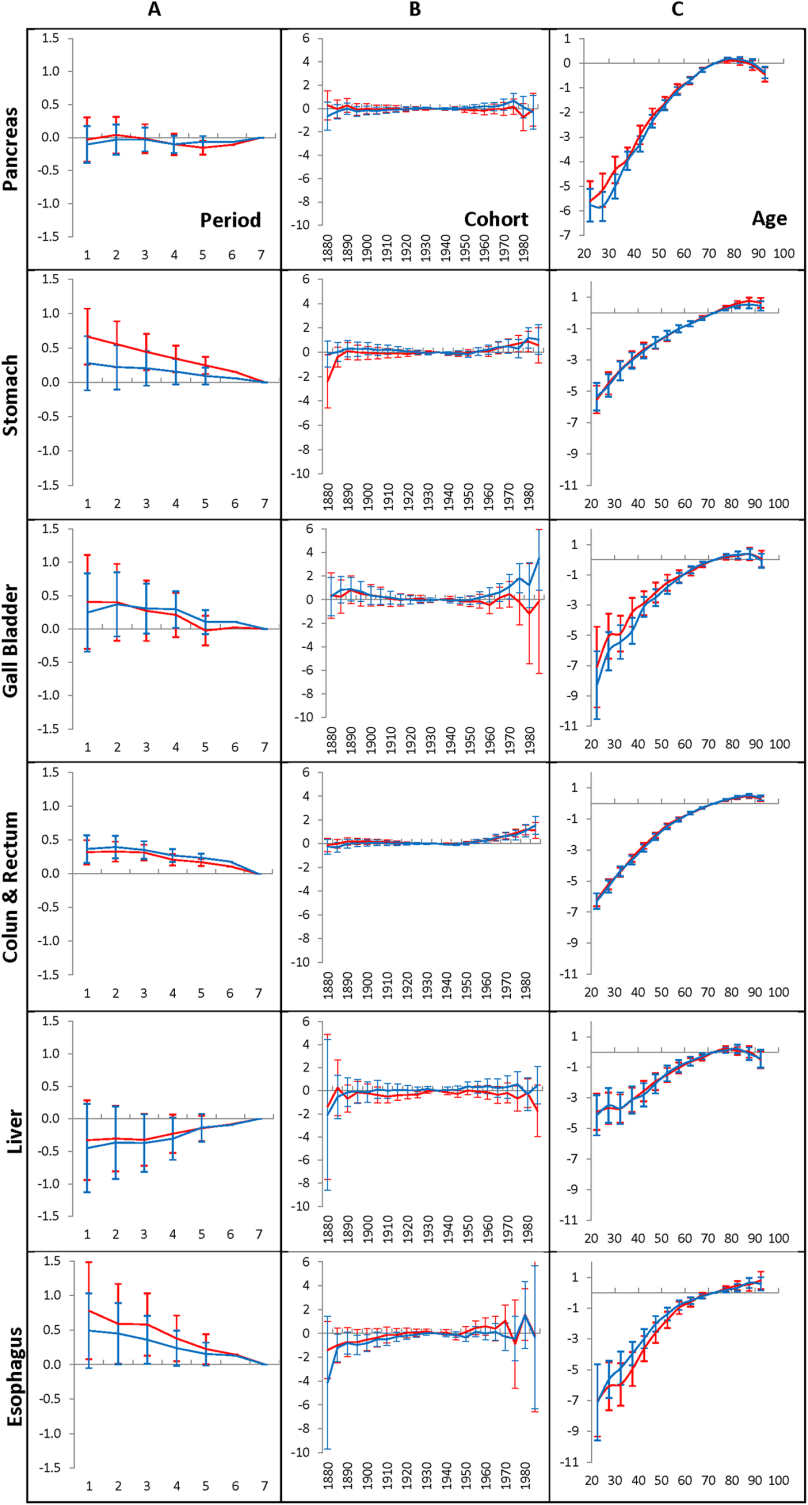
Time-period effects (A panel), birth-cohort effects (B panel) and age-at-diagnosis effects (C panel) for women who have lived in the Eastern (blue lines) and Western (red lines) geographic regions and diagnosed with GI cancers during 1975–2009.

**Fig 7:**
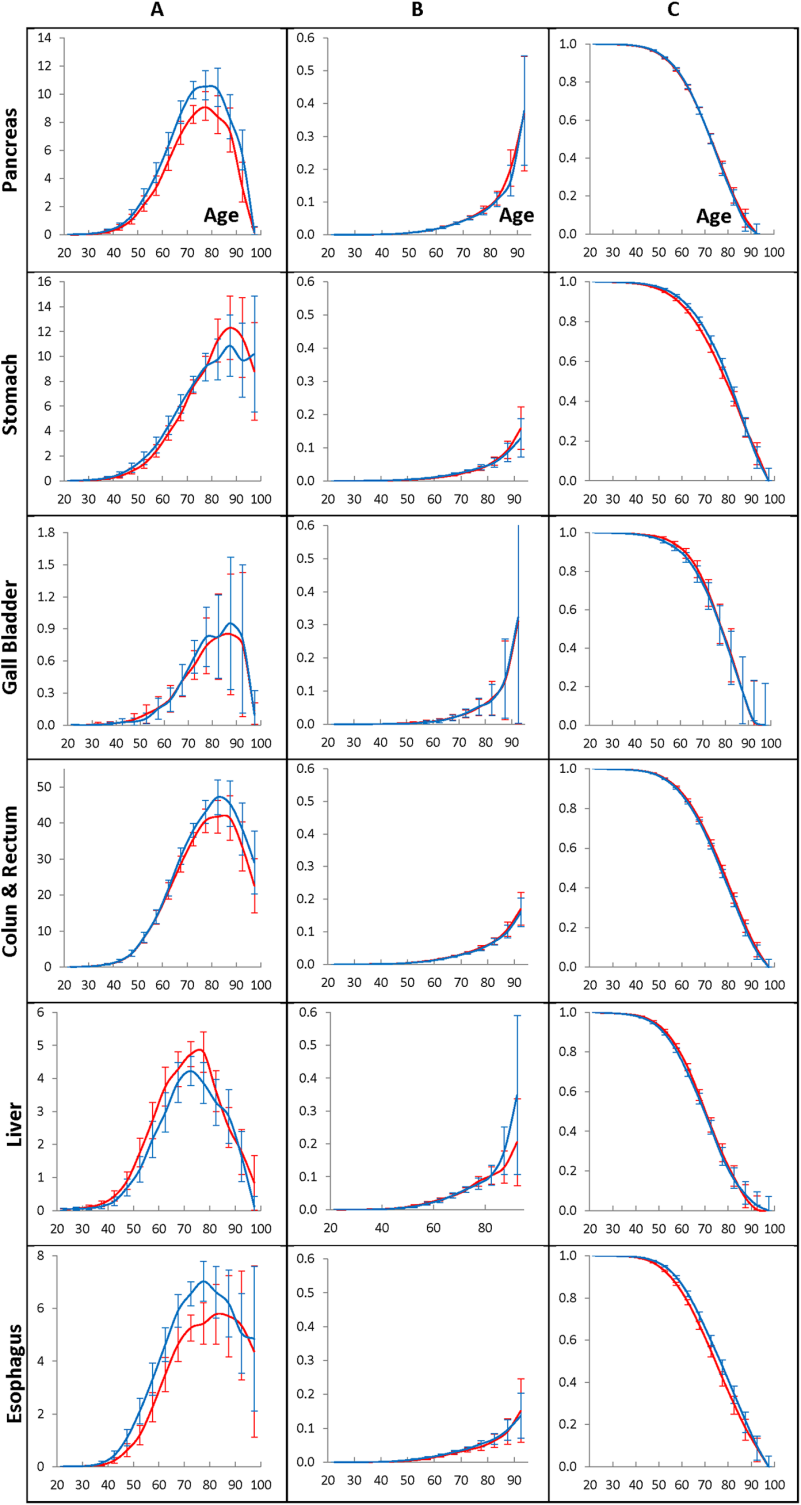
Population cancer hazard rates (A panel), individual cancer presentation rates (B panel), and individual cancer resistance rates (C panel) for men who have lived in the Eastern (blue lines) and Western (red lines) geographic regions and diagnosed with GI cancers during 1975–2009.

**Fig 8:**
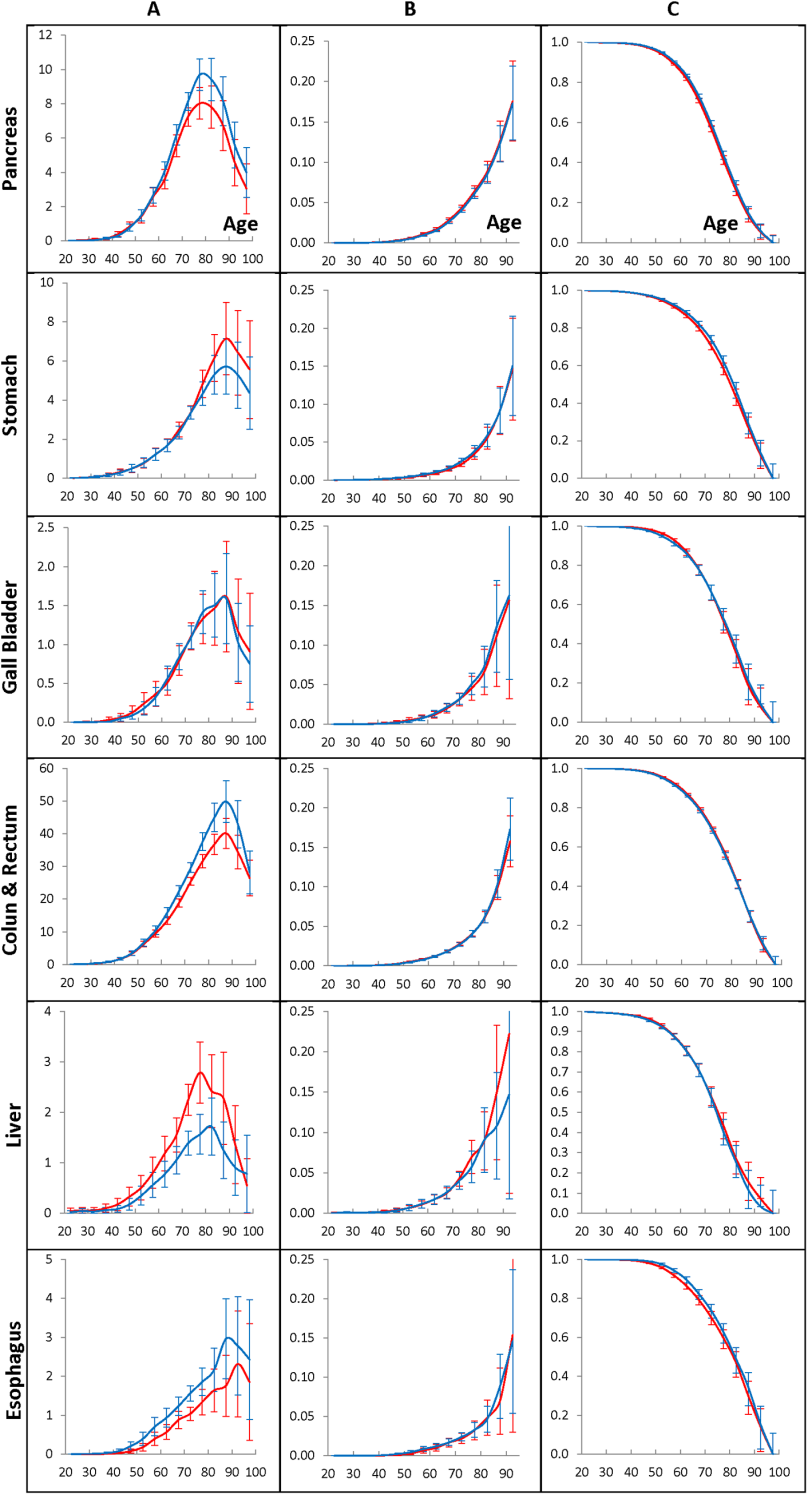
Population cancer hazard rates (A panel), individual cancer presentation rates (B panel), and individual cancer resistance rates (C panel) for women who have lived in the Eastern (blue lines) and Western (red lines) geographic regions and diagnosed with GI cancers during 1975–2009.

Outcomes from the second series of computational experiments (see the Materials and Methods section) are exhibited in Fig 5, Fig 6, Fig 7 and Fig 8. Fig 5 and Fig 6 show the time-period effects (A panels), the birth-cohort effects (B panels), and the age-at-diagnosis effects (C panels) of pancreatic, stomach, gallbladder, colon and rectum, liver and esophagus cancers in men who have lived during 1975–2009 in the Eastern (blue lines) and Western (red lines) regions. The Eastern region refers to the Atlanta, Connecticut, Detroit and Iowa areas, while the Western region refers to the San Francisco-Oakland SMSA, Seattle, Hawaii, New Mexico, and Utah areas. The effects are estimated by the time period of 2005–2009 and the age interval of 70–74, which are taken as anchors. The error bars indicate 95% of the confidence intervals of the corresponding effects. The time-period effects, the birth-cohort effects and age-at-diagnosis effects that related to the corresponding anchors, as well as the time period effects foregoing the anchored time period are presented without error bars (i.e. they are taken equal to zero). Fig 6 shows similar effects in women who have lived during 1975–2009 in the Eastern (blue lines) and Western (red lines) regions.

Fig 7 and Fig 8 show the population cancer hazard rates (A panels), the individual cancer presentation rates (B panels), and the individual cancer resistance rates (C panels) of the considered GI cancers in men (Fig 7) and in women (Fig 8) who lived in Eastern and Western regions during 1975–2009. The rates are estimated by the time period of 2005–2009 and the age interval of 70–74, taken as anchors. The error bars indicate 95% of the confidence intervals of the corresponding rates. On these figures, data for the Eastern and Western regions are shown by blue and red colors, correspondingly.

The performed computational experiments showed that the time-period effects of each of the considered GI cancers in men (Fig 5, A panels) and in women (Fig 6, A panels) who have lived in the Eastern and the Western regions are very similar. This is evidenced by the fact that the error bars of the corresponding time-period effects are greatly overlapping.

Our computational experiments also showed that the birth cohort effects of the pancreatic, stomach, gallbladder, liver, and esophagus cancers in men (Fig 5, B panels) and in women (Fig 6, B panels) living in the Eastern and the Western regions is negligible. This is clearly evidenced by the fact that the error bars of these effects contain the value of zero. However, for people who belong to several of the youngest cohorts, and who got the colon and rectum cancers, their birth cohort effects are small but significant. This is evidenced by the fact that the error bars of those effects do not include the value of zero.

The same experiments showed that the age effects strongly influence the occurrence of each of the considered GI cancers in men (Fig 5, C panels), as well in women (Fig 6, C panels). The age effects are sex- and cancer-specific, but are nearly independent of the geographic areas where cancer was diagnosed. This is evidenced by the fact that all error bars of the corresponding age effects determined for Western and Eastern regions are greatly overlapping.

The population cancer hazard rates of the considered cancers in men and women who have lived in Eastern and Western geographic areas are shown in the A panels of Fig 7 and Fig 8, correspondingly. These rates are anchored to the 2005–2009 time period. As can be seen from these figures, the population hazard rates in Western and Eastern regions have similar shapes, but the amplitudes of these shapes are dependent on the geographic areas of living cancer patients. It should be noted that the values of these rates are almost proportional to the ratios of their overall cumulative hazards, *H*_*UO*_, determined by formula (9).

For men, living in Eastern and Western regions, the individual cancer presentation rates and the individual cancer resistance rates of the considered cancers are shown in the B and C panels of Fig 7, correspondingly. These rates are anchored to the 2005–2009 time period. For women, the analogous rates are shown in the B and C panels of Fig 8. As can be seen from these panels, the error bars of the corresponding individual cancer presentation rates and individual cancer resistance rates are greatly overlapping, which suggests that these rates are invariant on the geographic areas.

### Dependency of the carcinogenic characteristics of the GI cancers upon sex

**Fig 9:**
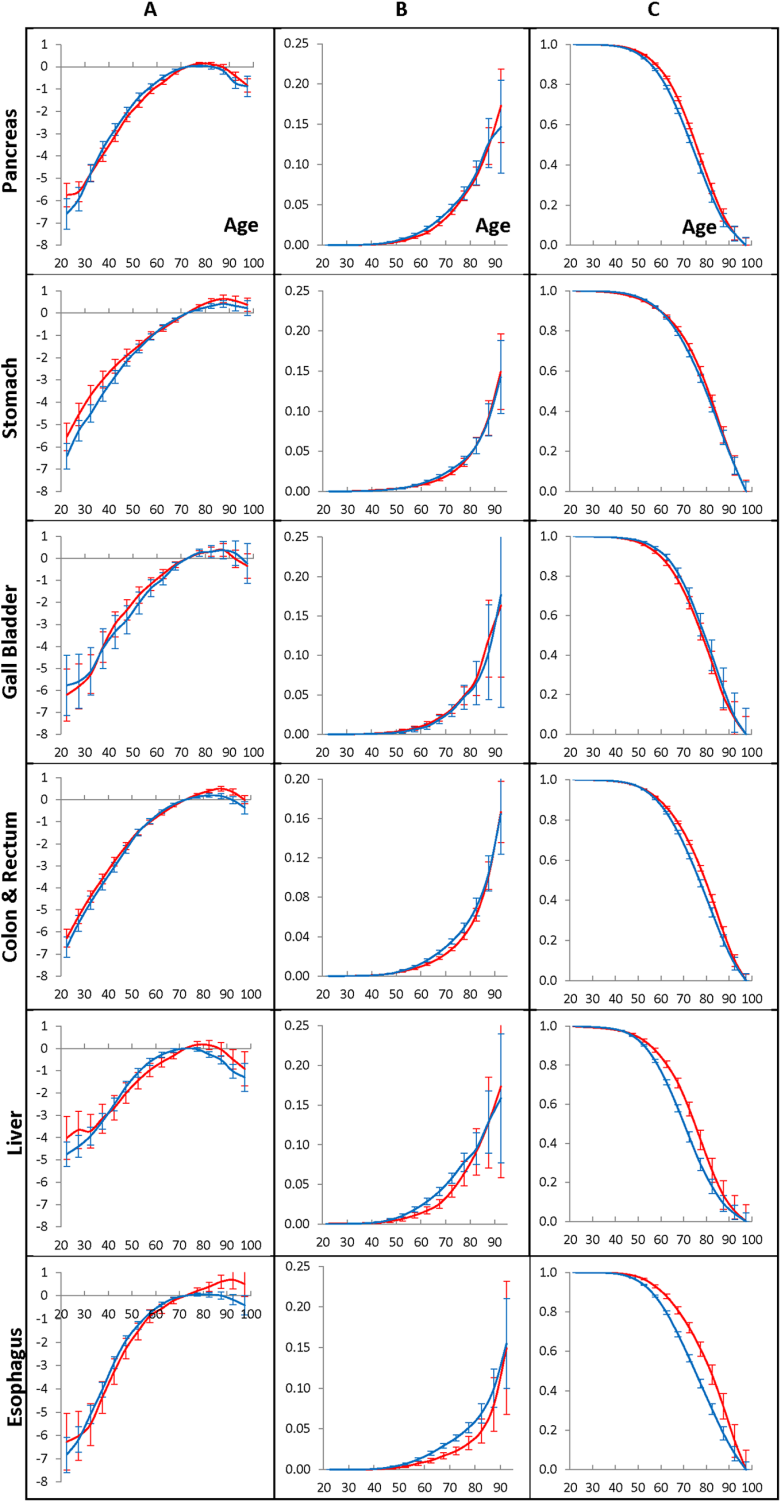
Age effects (A panel), individual cancer presentation rates (B panel) and individual cancer resistance rates (C panel) for men (blue lines) and women (red lines) diagnosed with GI cancers.

Overall, the performed computational experiments suggest that, for individuals susceptible to cancer, the age effects, the individual cancer presentation rates and the individual cancer resistance rates are: (i) intrinsic for each considered cancer, and (ii) invariant on the time period of cancer presentation and on the geographic areas where the cancer was diagnosed. However, these carcinogenic characteristics are dependent on sex. To demonstrate this, we compared the age effects in men and women who have lived during 1975–2009 in the Entire region and diagnosed with the considered GI cancers (see the A panels of Fig 9). These effects were determined by using the 2005–2009 time period and the age interval of 70–74 as anchors. The error bars indicate 95% of the confidence intervals of the corresponding effects. Analogously, comparisons of the individual cancer presentation rates and the individual cancer resistance rates in men with the similar rates in women are shown in the B and C panels of Fig 9, correspondingly. On Fig 9, data for men and women are shown in blue and red colors, correspondingly.

As can be seen from Fig 9 the patterns of the age effects, the individual cancer presentation rates and the individual cancer resistance rates are nearly the same in men and women, for the pancreatic, stomach, gallbladder and colon and rectum cancers. However, in cases of the liver and esophagus cancers the age effects in men and women are remarkably different from one another. For liver cancer, dependency of these characteristics on sex can be explained by the morphology and functionality of the liver which are modulated by sex hormones [24]. For esophagus cancer, the sex dependency of these characteristics can be explained by a larger histological heterogeneity of that cancer compared to other considered GI cancers. In fact, other considered cancers are mainly adenocarcinomas, while esophagus cancer has the two most prevalent histopathological subtypes, adenocarcinomas and squamous cell carcinoma. Therefore, the sex-specific variations in the mixture of these subtypes can differently influence the individual cancer presentation rates and the individual cancer resistance rates of esophagus cancer in men and women.

## Conclusions

Our computational experiments, performed on six different cancer sites from the GI system, allowed us to draw conclusions that, the age effects, the individual cancer presentation rates and the individual cancer resistance rates in aging are nearly independent of geographic areas of living of cancer patients and the time periods of cancer diagnosis. For the pancreatic, stomach, gallbladder, colon and rectum cancers, the patterns of these characteristics were nearly the same in men and women. However, for the liver and esophagus cancers, these patterns were remarkably different in men and women. For liver cancer, the sex dependency of these characteristics can be explained by the morphology and functionality of the liver which are modulated by sex hormones. For esophagus cancer, the sex dependency of these characteristics can be explained by the histological heterogeneity of that cancer.

The carcinogenic modeling performed in this work has the following limitations: (i) the data used is not categorized by many modifiable variables (such as lifestyle, dietary preferences, drinking and smoking habits), which influence cancer occurrence; (ii) the heterogeneity of the data used is not fully accounted (for instance, the data is not stratified by race, histopathological subtypes, and the stage at diagnosis); and (iii) the model used postulates a dichotomous susceptibility to cancer in a population. However, despite these limitations, we captured the base carcinogenic characteristics, intrinsic for each considered cancer site and offered explanations for the sex-specific divergences of those characteristics.

Based on the outcomes of our computational experiments, we concluded that there are three interrelated carcinogenic characteristics—the age effects, the individual cancer presentation rates and the individual cancer resistance rates—that are intrinsic for each considered cancer type. For a given organ site, an influence of sex (unmodifiable variable) on these carcinogenic characteristics can depend on differences in morphology and function of that organ in men and women. The age effects, as well as the individual cancer presentation rates and the individual cancer resistance rates should be further analyzed to better understand carcinogenesis in each organ site. Because these characteristics are invariant on modifiable risk factors of cancer, they can serve as baselines in predictive studies (by inferential statistics) and can be useful for the development of novel strategies for cancer prevention.

## Supporting Information

S1 Appendix Distribution of men and women populations and distribution of the GI cancer cases diagnosed in men and women who have lived in the Eastern and Western regions during 1975–2009. (DOC)
