# Supplementary material for: Invariant Characteristics of Carcinogenesis
Source: PLoS One. 2015 Oct 14;10(10):e0140405. doi: 10.1371/journal.pone.0140405 (PMC4605720; doi:10.1371/journal.pone.0140405)
Supplement: S1 Appendix — (DOC) [file pone.0140405.s001.doc]

**S1 Appendix. Distribution of men and women populations and distribution of the GI cancer cases diagnosed in men and women who have lived in the Eastern and Western regions during 1975-2009.**

**S1 Table 1. Distribution of the male populations () living in the Eastern region during seven time periods of 1975-2009**

| **Age** | | **Populations in the time periods** | | | | | | |
| --- | --- | --- | --- | --- | --- | --- | --- | --- |
| Index, $i$ | Interval | 1975-79 | 1980-84 | 1985-89 | 1990-94 | 1995-99 | 2000-04 | 2005-09 |
| 1 | 20-24 | 2535691 | 2635561 | 2445988 | 2234915 | 2069076 | 2190745 | 2257279 |
| 2 | 25-29 | 2404224 | 2582631 | 2677291 | 2492820 | 2403366 | 2209499 | 2219927 |
| 3 | 30-34 | 2051569 | 2357003 | 2592003 | 2731517 | 2621647 | 2496173 | 2209014 |
| 4 | 35-39 | 1632778 | 1946022 | 2310251 | 2591314 | 2778793 | 2622863 | 2431060 |
| 5 | 40-44 | 1463255 | 1574905 | 1930811 | 2317444 | 2585071 | 2721604 | 2561702 |
| 6 | 45-49 | 1528765 | 1390071 | 1549050 | 1877954 | 2260133 | 2503390 | 2628528 |
| 7 | 50-54 | 1589879 | 1447091 | 1335352 | 1470120 | 1818730 | 2173820 | 2406726 |
| 8 | 55-59 | 1448103 | 1433400 | 1317755 | 1235183 | 1378914 | 1688938 | 2037194 |
| 9 | 60-64 | 1183116 | 1243912 | 1250887 | 1179789 | 1108973 | 1226096 | 1527366 |
| 10 | 65-69 | 889163 | 984601 | 1051321 | 1060956 | 996574 | 944888 | 1080528 |
| 11 | 70-74 | 637922 | 701143 | 772945 | 844873 | 872708 | 838939 | 807889 |
| 12 | 75-79 | 422861 | 464289 | 523040 | 594976 | 665083 | 697388 | 676274 |
| 13 | 80-84 | 263274 | 264564 | 291326 | 337388 | 400833 | 461097 | 503727 |
| 14 | 85-89 | 118372 | 134090 | 141406 | 157408 | 185053 | 217301 | 265102 |
| 15 | 90-94 | 39961 | 45267 | 47737 | 53139 | 62472 | 73359 | 89495 |
| 16 | 95-99 | 8672 | 9824 | 10360 | 11532 | 13558 | 15920 | 19423 |

**S1 Table 2. Distribution of the male populations () living in the Western region during seven time periods of 1975-2009**

| **Age** | | **Populations in the time periods** | | | | | | |
| --- | --- | --- | --- | --- | --- | --- | --- | --- |
| Index, $i$ | Interval | 1975-79 | 1980-84 | 1985-89 | 1990-94 | 1995-99 | 2000-04 | 2005-09 |
| 1 | 20-24 | 2287757 | 2431352 | 2294373 | 2233664 | 2276122 | 2603927 | 2693702 |
| 2 | 25-29 | 2152893 | 2524938 | 2637329 | 2544750 | 2516251 | 2512191 | 2751568 |
| 3 | 30-34 | 1802514 | 2290579 | 2582285 | 2787514 | 2751454 | 2655086 | 2576969 |
| 4 | 35-39 | 1398934 | 1818887 | 2267775 | 2633364 | 2823312 | 2660976 | 2622567 |
| 5 | 40-44 | 1192589 | 1405107 | 1832559 | 2329427 | 2614518 | 2725590 | 2615414 |
| 6 | 45-49 | 1159573 | 1183912 | 1395375 | 1814943 | 2269702 | 2543531 | 2679801 |
| 7 | 50-54 | 1167768 | 1149167 | 1159743 | 1381352 | 1816560 | 2259085 | 2504800 |
| 8 | 55-59 | 1069751 | 1130481 | 1095371 | 1124847 | 1340298 | 1744861 | 2196832 |
| 9 | 60-64 | 895221 | 1008055 | 1038498 | 1037523 | 1075399 | 1261931 | 1663498 |
| 10 | 65-69 | 706281 | 804147 | 912271 | 953516 | 961437 | 982811 | 1179241 |
| 11 | 70-74 | 496977 | 590464 | 672910 | 776894 | 822085 | 835238 | 882514 |
| 12 | 75-79 | 313169 | 379036 | 458996 | 541591 | 637194 | 683424 | 699057 |
| 13 | 80-84 | 177044 | 205176 | 251269 | 313517 | 385635 | 459549 | 506318 |
| 14 | 85-89 | 84031 | 95539 | 111199 | 137138 | 177877 | 218360 | 274593 |
| 15 | 90-94 | 29071 | 33053 | 38471 | 47444 | 61539 | 75544 | 94999 |
| 16 | 95-99 | 6482 | 7370 | 8578 | 10579 | 13722 | 16845 | 21182 |

**S1 Table 3. Distribution of the female populations () living in the Eastern region during seven time periods of 1975-2009**

| **Age** | | **Populations in the time periods** | | | | | | |
| --- | --- | --- | --- | --- | --- | --- | --- | --- |
| Index, $i$ | Interval | 1975-79 | 1980-84 | 1985-89 | 1990-94 | 1995-99 | 2000-04 | 2005-09 |
| 1 | 20-24 | 2646223 | 2715988 | 2495233 | 2253885 | 2048105 | 2125478 | 2181625 |
| 2 | 25-29 | 2471581 | 2645478 | 2740074 | 2548241 | 2430600 | 2213517 | 2244673 |
| 3 | 30-34 | 2128818 | 2456703 | 2684545 | 2802883 | 2654192 | 2517880 | 2269568 |
| 4 | 35-39 | 1712139 | 2025674 | 2406836 | 2678230 | 2840955 | 2672241 | 2503271 |
| 5 | 40-44 | 1547101 | 1668528 | 2040329 | 2432447 | 2685421 | 2805398 | 2645881 |
| 6 | 45-49 | 1620805 | 1480147 | 1625374 | 1955249 | 2352999 | 2604577 | 2744784 |
| 7 | 50-54 | 1704643 | 1539016 | 1420268 | 1560014 | 1921082 | 2293686 | 2528650 |
| 8 | 55-59 | 1578566 | 1577296 | 1442739 | 1341707 | 1470208 | 1799216 | 2186913 |
| 9 | 60-64 | 1344233 | 1422303 | 1428921 | 1325226 | 1238609 | 1348719 | 1674581 |
| 10 | 65-69 | 1128729 | 1236311 | 1311236 | 1305822 | 1208958 | 1128152 | 1241698 |
| 11 | 70-74 | 911024 | 1000322 | 1086867 | 1156505 | 1159912 | 1085546 | 1025953 |
| 12 | 75-79 | 700821 | 777825 | 858376 | 943433 | 1012621 | 1011465 | 945421 |
| 13 | 80-84 | 498168 | 531656 | 591493 | 663316 | 740684 | 798417 | 817070 |
| 14 | 85-89 | 235546 | 295398 | 338519 | 389683 | 444351 | 483987 | 538209 |
| 15 | 90-94 | 107580 | 134917 | 154611 | 177979 | 202948 | 221051 | 245815 |
| 16 | 95-99 | 32044 | 40186 | 46052 | 53013 | 60450 | 65842 | 73218 |

**S1 Table 4. Distribution of the female populations () living in the Western region during seven time periods of 1975-2009**

| **Age** | | **Populations in the time periods** | | | | | | |
| --- | --- | --- | --- | --- | --- | --- | --- | --- |
| Index, $i$ | Interval | 1975-79 | 1980-84 | 1985-89 | 1990-94 | 1995-99 | 2000-04 | 2005-09 |
| 1 | 20-24 | 2171895 | 2306814 | 2137981 | 2083485 | 2152226 | 2464076 | 2522807 |
| 2 | 25-29 | 2104569 | 2453719 | 2530652 | 2435716 | 2408639 | 2419379 | 2690227 |
| 3 | 30-34 | 1792733 | 2263258 | 2534476 | 2701161 | 2640146 | 2532287 | 2489984 |
| 4 | 35-39 | 1377874 | 1794183 | 2246907 | 2591630 | 2755128 | 2590317 | 2560315 |
| 5 | 40-44 | 1187875 | 1398533 | 1830762 | 2326282 | 2605639 | 2708609 | 2582410 |
| 6 | 45-49 | 1181734 | 1187415 | 1392158 | 1818044 | 2304457 | 2576851 | 2689772 |
| 7 | 50-54 | 1233217 | 1201557 | 1188581 | 1404478 | 1856465 | 2321041 | 2570018 |
| 8 | 55-59 | 1135666 | 1217697 | 1161237 | 1176350 | 1391462 | 1812832 | 2296946 |
| 9 | 60-64 | 960625 | 1098718 | 1162380 | 1150592 | 1162797 | 1341575 | 1770248 |
| 10 | 65-69 | 822902 | 941644 | 1060654 | 1129023 | 1098752 | 1100022 | 1298667 |
| 11 | 70-74 | 639240 | 748423 | 848647 | 958135 | 1023647 | 1006451 | 1027365 |
| 12 | 75-79 | 471786 | 555330 | 651057 | 746052 | 858235 | 919426 | 892112 |
| 13 | 80-84 | 325506 | 367162 | 431559 | 511070 | 596046 | 688306 | 749145 |
| 14 | 85-89 | 157793 | 201358 | 238064 | 284712 | 347426 | 402323 | 471669 |
| 15 | 90-94 | 70176 | 89551 | 105876 | 126621 | 154513 | 178927 | 209768 |
| 16 | 95-99 | 20429 | 26069 | 30821 | 36860 | 44980 | 52087 | 61065 |

**S1 Table 5. Distribution of the number of occurrences () of pancreatic cancer in men living in the Eastern** region during seven time periods of 1975-2009

| **Age** | | **Number of cancers in the time periods** | | | | | | |
| --- | --- | --- | --- | --- | --- | --- | --- | --- |
| Index, | Interval | 1975-79 | 1980-84 | 1985-89 | 1990-94 | 1995-99 | 2000-04 | 2005-09 |
| 1 | 20-24 | 3 | 3 | 0 | 1 | 3 | 0 | 0 |
| 2 | 25-29 | 5 | 1 | 4 | 1 | 7 | 3 | 5 |
| 3 | 30-34 | 10 | 8 | 10 | 11 | 13 | 12 | 18 |
| 4 | 35-39 | 17 | 24 | 34 | 32 | 30 | 40 | 32 |
| 5 | 40-44 | 41 | 44 | 59 | 56 | 72 | 88 | 71 |
| 6 | 45-49 | 105 | 94 | 89 | 84 | 145 | 160 | 172 |
| 7 | 50-54 | 181 | 180 | 152 | 171 | 228 | 278 | 295 |
| 8 | 55-59 | 279 | 304 | 243 | 242 | 264 | 358 | 416 |
| 9 | 60-64 | 366 | 362 | 386 | 292 | 333 | 360 | 495 |
| 10 | 65-69 | 351 | 378 | 406 | 450 | 367 | 375 | 487 |
| 11 | 70-74 | 286 | 346 | 379 | 421 | 386 | 405 | 397 |
| 12 | 75-79 | 218 | 239 | 248 | 302 | 316 | 332 | 371 |
| 13 | 80-84 | 115 | 133 | 168 | 145 | 186 | 234 | 275 |
| 14 | 85-89 | 49 | 58 | 68 | 50 | 68 | 80 | 115 |
| 15 | 90-94 | 9 | 10 | 18 | 13 | 17 | 17 | 28 |
| 16 | 95-99 | 1 | 1 | 5 | 2 | 1 | 1 | 5 |

**S1 Table 6. Distribution of the number of occurrences () of pancreatic cancer in men living in the Western** region during seven time periods of 1975-2009

| **Age** | | **Number of cancers in the time periods** | | | | | | |
| --- | --- | --- | --- | --- | --- | --- | --- | --- |
| Index, | Interval | 1975-79 | 1980-84 | 1985-89 | 1990-94 | 1995-99 | 2000-04 | 2005-09 |
| 1 | 20-24 | 2 | 1 | 0 | 0 | 0 | 1 | 0 |
| 2 | 25-29 | 3 | 2 | 1 | 1 | 0 | 3 | 5 |
| 3 | 30-34 | 3 | 6 | 11 | 11 | 4 | 7 | 9 |
| 4 | 35-39 | 15 | 22 | 19 | 21 | 24 | 24 | 25 |
| 5 | 40-44 | 33 | 45 | 33 | 61 | 55 | 51 | 63 |
| 6 | 45-49 | 61 | 76 | 75 | 85 | 106 | 123 | 137 |
| 7 | 50-54 | 141 | 140 | 122 | 115 | 171 | 228 | 289 |
| 8 | 55-59 | 212 | 212 | 173 | 168 | 206 | 270 | 364 |
| 9 | 60-64 | 283 | 297 | 277 | 237 | 252 | 312 | 414 |
| 10 | 65-69 | 309 | 293 | 313 | 328 | 309 | 320 | 437 |
| 11 | 70-74 | 272 | 263 | 299 | 302 | 330 | 312 | 364 |
| 12 | 75-79 | 191 | 197 | 231 | 252 | 223 | 285 | 315 |
| 13 | 80-84 | 84 | 99 | 112 | 122 | 156 | 176 | 231 |
| 14 | 85-89 | 46 | 45 | 58 | 43 | 51 | 67 | 111 |
| 15 | 90-94 | 9 | 13 | 4 | 9 | 12 | 9 | 14 |
| 16 | 95-99 | 2 | 1 | 2 | 0 | 2 | 1 | 6 |

**S1 Table 7. Distribution of the number of occurrences () of stomach cancer in men living in the Eastern** region during seven time periods of 1975-2009

| **Age** | | **Number of cancers in the time periods** | | | | | | |
| --- | --- | --- | --- | --- | --- | --- | --- | --- |
| Index, | Interval | 1975-79 | 1980-84 | 1985-89 | 1990-94 | 1995-99 | 2000-04 | 2005-09 |
| 1 | 20-24 | 3 | 1 | 2 | 5 | 2 | 5 | 0 |
| 2 | 25-29 | 9 | 13 | 2 | 9 | 9 | 13 | 12 |
| 3 | 30-34 | 17 | 15 | 25 | 24 | 13 | 19 | 27 |
| 4 | 35-39 | 26 | 35 | 31 | 40 | 43 | 43 | 49 |
| 5 | 40-44 | 74 | 48 | 69 | 85 | 80 | 69 | 73 |
| 6 | 45-49 | 125 | 111 | 115 | 114 | 115 | 148 | 170 |
| 7 | 50-54 | 213 | 182 | 169 | 172 | 187 | 212 | 229 |
| 8 | 55-59 | 332 | 301 | 273 | 237 | 224 | 246 | 277 |
| 9 | 60-64 | 437 | 415 | 403 | 309 | 282 | 296 | 348 |
| 10 | 65-69 | 429 | 530 | 468 | 440 | 350 | 313 | 313 |
| 11 | 70-74 | 376 | 453 | 472 | 482 | 405 | 335 | 298 |
| 12 | 75-79 | 352 | 333 | 400 | 351 | 414 | 320 | 314 |
| 13 | 80-84 | 247 | 238 | 245 | 224 | 234 | 276 | 234 |
| 14 | 85-89 | 111 | 144 | 125 | 154 | 136 | 111 | 149 |
| 15 | 90-94 | 39 | 38 | 46 | 51 | 38 | 36 | 45 |
| 16 | 95-99 | 6 | 14 | 12 | 8 | 11 | 7 | 6 |

**S1 Table 8. Distribution of the number of occurrences () of stomach cancer in men living in the Western** region during seven time periods of 1975-2009

| **Age** | | **Number of cancers in the time periods** | | | | | | |
| --- | --- | --- | --- | --- | --- | --- | --- | --- |
| Index, | Interval | 1975-79 | 1980-84 | 1985-89 | 1990-94 | 1995-99 | 2000-04 | 2005-09 |
| 1 | 20-24 | 0 | 5 | 5 | 5 | 4 | 2 | 4 |
| 2 | 25-29 | 9 | 13 | 1 | 7 | 10 | 11 | 9 |
| 3 | 30-34 | 6 | 13 | 24 | 18 | 27 | 19 | 20 |
| 4 | 35-39 | 37 | 26 | 33 | 44 | 47 | 42 | 42 |
| 5 | 40-44 | 44 | 54 | 70 | 77 | 78 | 68 | 69 |
| 6 | 45-49 | 106 | 89 | 94 | 96 | 125 | 141 | 144 |
| 7 | 50-54 | 197 | 179 | 143 | 131 | 137 | 180 | 213 |
| 8 | 55-59 | 244 | 272 | 249 | 198 | 219 | 258 | 281 |
| 9 | 60-64 | 358 | 368 | 377 | 342 | 287 | 259 | 285 |
| 10 | 65-69 | 369 | 410 | 398 | 403 | 360 | 336 | 328 |
| 11 | 70-74 | 366 | 377 | 455 | 468 | 420 | 361 | 335 |
| 12 | 75-79 | 297 | 311 | 359 | 360 | 366 | 364 | 317 |
| 13 | 80-84 | 200 | 253 | 224 | 247 | 269 | 305 | 264 |
| 14 | 85-89 | 95 | 109 | 114 | 122 | 124 | 166 | 159 |
| 15 | 90-94 | 32 | 38 | 36 | 43 | 47 | 42 | 49 |
| 16 | 95-99 | 6 | 8 | 5 | 9 | 9 | 5 | 5 |

**S1 Table 9. Distribution of the number of occurrences () of gallbladder cancer in men living in the Eastern** region during seven time periods of 1975-2009

| **Age** | | **Number of cancers in the time periods** | | | | | | |
| --- | --- | --- | --- | --- | --- | --- | --- | --- |
| Index, | Interval | 1975-79 | 1980-84 | 1985-89 | 1990-94 | 1995-99 | 2000-04 | 2005-09 |
| 1 | 20-24 | 0 | 0 | 0 | 1 | 1 | 0 | 0 |
| 2 | 25-29 | 0 | 0 | 0 | 0 | 0 | 1 | 1 |
| 3 | 30-34 | 1 | 0 | 0 | 0 | 1 | 0 | 2 |
| 4 | 35-39 | 0 | 2 | 0 | 3 | 3 | 2 | 1 |
| 5 | 40-44 | 0 | 3 | 5 | 5 | 0 | 6 | 6 |
| 6 | 45-49 | 1 | 3 | 1 | 3 | 9 | 6 | 8 |
| 7 | 50-54 | 9 | 6 | 8 | 3 | 9 | 10 | 11 |
| 8 | 55-59 | 24 | 15 | 20 | 5 | 16 | 13 | 22 |
| 9 | 60-64 | 25 | 21 | 26 | 16 | 17 | 18 | 17 |
| 10 | 65-69 | 35 | 32 | 33 | 29 | 27 | 20 | 30 |
| 11 | 70-74 | 44 | 54 | 34 | 28 | 33 | 28 | 24 |
| 12 | 75-79 | 36 | 40 | 28 | 28 | 31 | 32 | 44 |
| 13 | 80-84 | 21 | 20 | 22 | 15 | 25 | 26 | 24 |
| 14 | 85-89 | 19 | 22 | 15 | 10 | 9 | 11 | 6 |
| 15 | 90-94 | 2 | 8 | 3 | 2 | 4 | 4 | 4 |
| 16 | 95-99 | 1 | 0 | 0 | 2 | 1 | 0 | 1 |

**S1 Table 10. Distribution of the number of occurrences () of gallbladder cancer in men living in the Western** region during seven time periods of 1975-2009

| **Age** | | **Number of cancers in the time periods** | | | | | | |
| --- | --- | --- | --- | --- | --- | --- | --- | --- |
| Index, | Interval | 1975-79 | 1980-84 | 1985-89 | 1990-94 | 1995-99 | 2000-04 | 2005-09 |
| 1 | 20-24 | 0 | 0 | 0 | 0 | 0 | 0 | 1 |
| 2 | 25-29 | 0 | 0 | 0 | 0 | 0 | 1 | 0 |
| 3 | 30-34 | 0 | 2 | 0 | 0 | 2 | 0 | 0 |
| 4 | 35-39 | 0 | 0 | 1 | 2 | 1 | 2 | 7 |
| 5 | 40-44 | 1 | 0 | 4 | 1 | 4 | 4 | 3 |
| 6 | 45-49 | 7 | 3 | 1 | 2 | 9 | 2 | 6 |
| 7 | 50-54 | 14 | 7 | 8 | 9 | 8 | 7 | 10 |
| 8 | 55-59 | 10 | 12 | 12 | 11 | 14 | 16 | 14 |
| 9 | 60-64 | 17 | 19 | 12 | 14 | 18 | 16 | 21 |
| 10 | 65-69 | 26 | 27 | 26 | 25 | 20 | 20 | 27 |
| 11 | 70-74 | 30 | 22 | 22 | 33 | 28 | 28 | 22 |
| 12 | 75-79 | 22 | 33 | 34 | 26 | 19 | 25 | 23 |
| 13 | 80-84 | 21 | 17 | 17 | 23 | 14 | 14 | 26 |
| 14 | 85-89 | 9 | 7 | 9 | 6 | 12 | 11 | 14 |
| 15 | 90-94 | 1 | 4 | 3 | 2 | 3 | 4 | 4 |
| 16 | 95-99 | 0 | 0 | 0 | 1 | 1 | 0 | 1 |

**S1 Table 11. Distribution of the number of occurrences () of colon and rectum cancer in men living in the Eastern** region during seven time periods of 1975-2009

| **Age** | | **Number of cancers in the time periods** | | | | | | |
| --- | --- | --- | --- | --- | --- | --- | --- | --- |
| Index, | Interval | 1975-79 | 1980-84 | 1985-89 | 1990-94 | 1995-99 | 2000-04 | 2005-09 |
| 1 | 20-24 | 9 | 18 | 13 | 13 | 13 | 21 | 20 |
| 2 | 25-29 | 32 | 39 | 31 | 40 | 38 | 47 | 51 |
| 3 | 30-34 | 68 | 62 | 76 | 81 | 90 | 114 | 101 |
| 4 | 35-39 | 106 | 105 | 138 | 185 | 194 | 234 | 207 |
| 5 | 40-44 | 201 | 228 | 252 | 290 | 365 | 434 | 421 |
| 6 | 45-49 | 458 | 388 | 458 | 513 | 585 | 714 | 803 |
| 7 | 50-54 | 908 | 847 | 755 | 762 | 998 | 1334 | 1420 |
| 8 | 55-59 | 1421 | 1549 | 1383 | 1228 | 1286 | 1466 | 1586 |
| 9 | 60-64 | 1888 | 2163 | 2199 | 1866 | 1662 | 1729 | 1629 |
| 10 | 65-69 | 2130 | 2497 | 2677 | 2540 | 2137 | 1817 | 1674 |
| 11 | 70-74 | 2110 | 2347 | 2639 | 2510 | 2383 | 2023 | 1516 |
| 12 | 75-79 | 1631 | 1939 | 2208 | 2126 | 2082 | 2063 | 1387 |
| 13 | 80-84 | 1174 | 1226 | 1452 | 1494 | 1498 | 1446 | 1207 |
| 14 | 85-89 | 581 | 656 | 668 | 651 | 729 | 712 | 671 |
| 15 | 90-94 | 144 | 195 | 191 | 214 | 218 | 226 | 206 |
| 16 | 95-99 | 16 | 25 | 49 | 35 | 23 | 44 | 33 |

**S1 Table 12. Distribution of the number of occurrences () of colon and rectum cancer in men living in the Western** region during seven time periods of 1975-2009

| **Age** | | **Number of cancers in the time periods** | | | | | | |
| --- | --- | --- | --- | --- | --- | --- | --- | --- |
| Index, | Interval | 1975-79 | 1980-84 | 1985-89 | 1990-94 | 1995-99 | 2000-04 | 2005-09 |
| 1 | 20-24 | 14 | 12 | 16 | 8 | 18 | 20 | 30 |
| 2 | 25-29 | 34 | 25 | 41 | 28 | 39 | 42 | 68 |
| 3 | 30-34 | 56 | 55 | 72 | 102 | 88 | 87 | 127 |
| 4 | 35-39 | 98 | 112 | 123 | 138 | 168 | 203 | 226 |
| 5 | 40-44 | 155 | 164 | 188 | 271 | 316 | 365 | 380 |
| 6 | 45-49 | 310 | 295 | 354 | 428 | 582 | 681 | 761 |
| 7 | 50-54 | 609 | 576 | 623 | 708 | 855 | 1188 | 1425 |
| 8 | 55-59 | 953 | 1036 | 1053 | 1023 | 1144 | 1383 | 1624 |
| 9 | 60-64 | 1285 | 1452 | 1687 | 1436 | 1404 | 1503 | 1673 |
| 10 | 65-69 | 1422 | 1661 | 1958 | 1896 | 1765 | 1677 | 1643 |
| 11 | 70-74 | 1403 | 1710 | 1945 | 1926 | 1837 | 1719 | 1529 |
| 12 | 75-79 | 1087 | 1328 | 1686 | 1727 | 1710 | 1574 | 1373 |
| 13 | 80-84 | 718 | 859 | 951 | 1092 | 1143 | 1160 | 1076 |
| 14 | 85-89 | 366 | 451 | 494 | 478 | 547 | 637 | 557 |
| 15 | 90-94 | 114 | 138 | 165 | 142 | 170 | 173 | 178 |
| 16 | 95-99 | 12 | 24 | 30 | 19 | 29 | 28 | 27 |

**S1 Table 13. Distribution of the number of occurrences () of liver cancer in men living in the Eastern** region during seven time periods of 1975-2009

| **Age** | | **Number of cancers in the time periods** | | | | | | |
| --- | --- | --- | --- | --- | --- | --- | --- | --- |
| Index, | Interval | 1975-79 | 1980-84 | 1985-89 | 1990-94 | 1995-99 | 2000-04 | 2005-09 |
| 1 | 20-24 | 7 | 3 | 2 | 4 | 4 | 5 | 7 |
| 2 | 25-29 | 2 | 4 | 7 | 4 | 3 | 10 | 12 |
| 3 | 30-34 | 1 | 6 | 9 | 9 | 8 | 13 | 9 |
| 4 | 35-39 | 6 | 9 | 11 | 19 | 23 | 13 | 21 |
| 5 | 40-44 | 12 | 10 | 15 | 40 | 51 | 45 | 40 |
| 6 | 45-49 | 30 | 17 | 33 | 55 | 109 | 151 | 121 |
| 7 | 50-54 | 46 | 35 | 41 | 56 | 99 | 238 | 316 |
| 8 | 55-59 | 75 | 69 | 75 | 91 | 119 | 228 | 403 |
| 9 | 60-64 | 88 | 93 | 149 | 109 | 127 | 166 | 287 |
| 10 | 65-69 | 102 | 109 | 164 | 156 | 158 | 143 | 222 |
| 11 | 70-74 | 79 | 92 | 107 | 154 | 160 | 183 | 179 |
| 12 | 75-79 | 56 | 57 | 86 | 97 | 132 | 154 | 138 |
| 13 | 80-84 | 40 | 44 | 46 | 52 | 76 | 71 | 88 |
| 14 | 85-89 | 13 | 11 | 19 | 21 | 33 | 38 | 59 |
| 15 | 90-94 | 2 | 7 | 2 | 2 | 6 | 7 | 11 |
| 16 | 95-99 | 0 | 2 | 0 | 0 | 2 | 2 | 1 |

**S1 Table 14. Distribution of the number of occurrences () of liver cancer in men living in the Western** region during seven time periods of 1975-2009

| **Age** | | **Number of cancers in the time periods** | | | | | | |
| --- | --- | --- | --- | --- | --- | --- | --- | --- |
| Index, | Interval | 1975-79 | 1980-84 | 1985-89 | 1990-94 | 1995-99 | 2000-04 | 2005-09 |
| 1 | 20-24 | 3 | 3 | 0 | 4 | 3 | 4 | 6 |
| 2 | 25-29 | 6 | 4 | 5 | 6 | 6 | 5 | 9 |
| 3 | 30-34 | 9 | 9 | 13 | 15 | 9 | 15 | 7 |
| 4 | 35-39 | 10 | 13 | 25 | 24 | 26 | 27 | 32 |
| 5 | 40-44 | 12 | 19 | 31 | 58 | 77 | 59 | 55 |
| 6 | 45-49 | 34 | 33 | 31 | 60 | 129 | 169 | 143 |
| 7 | 50-54 | 53 | 61 | 71 | 85 | 131 | 268 | 361 |
| 8 | 55-59 | 72 | 89 | 105 | 102 | 159 | 236 | 446 |
| 9 | 60-64 | 99 | 109 | 121 | 138 | 189 | 213 | 321 |
| 10 | 65-69 | 88 | 99 | 124 | 150 | 200 | 184 | 258 |
| 11 | 70-74 | 97 | 105 | 96 | 140 | 187 | 172 | 204 |
| 12 | 75-79 | 70 | 79 | 91 | 116 | 136 | 144 | 160 |
| 13 | 80-84 | 34 | 39 | 41 | 48 | 64 | 74 | 99 |
| 14 | 85-89 | 9 | 10 | 20 | 25 | 21 | 21 | 37 |
| 15 | 90-94 | 4 | 6 | 5 | 4 | 7 | 9 | 4 |
| 16 | 95-99 | 0 | 1 | 0 | 0 | 1 | 1 | 0 |

**S1 Table 15. Distribution of the number of occurrences () of esophagus cancer in men living in the Eastern** region during seven time periods of 1975-2009

| **Age** | | **Number of cancers in the time periods** | | | | | | |
| --- | --- | --- | --- | --- | --- | --- | --- | --- |
| Index, | Interval | 1975-79 | 1980-84 | 1985-89 | 1990-94 | 1995-99 | 2000-04 | 2005-09 |
| 1 | 20-24 | 0 | 0 | 1 | 0 | 2 | 1 | 0 |
| 2 | 25-29 | 2 | 3 | 1 | 2 | 2 | 2 | 2 |
| 3 | 30-34 | 2 | 4 | 6 | 3 | 4 | 8 | 2 |
| 4 | 35-39 | 10 | 10 | 16 | 19 | 15 | 21 | 20 |
| 5 | 40-44 | 36 | 37 | 38 | 47 | 58 | 48 | 46 |
| 6 | 45-49 | 85 | 75 | 94 | 90 | 136 | 114 | 124 |
| 7 | 50-54 | 177 | 171 | 142 | 148 | 199 | 240 | 195 |
| 8 | 55-59 | 231 | 253 | 205 | 221 | 222 | 308 | 329 |
| 9 | 60-64 | 265 | 278 | 308 | 286 | 246 | 301 | 351 |
| 10 | 65-69 | 232 | 258 | 312 | 309 | 305 | 303 | 320 |
| 11 | 70-74 | 205 | 184 | 254 | 265 | 301 | 279 | 270 |
| 12 | 75-79 | 123 | 159 | 145 | 221 | 223 | 258 | 228 |
| 13 | 80-84 | 61 | 60 | 79 | 96 | 136 | 164 | 172 |
| 14 | 85-89 | 31 | 29 | 42 | 34 | 50 | 66 | 78 |
| 15 | 90-94 | 5 | 6 | 11 | 13 | 16 | 10 | 23 |
| 16 | 95-99 | 1 | 2 | 1 | 1 | 1 | 6 | 2 |

**S1 Table 16. Distribution of the number of occurrences () of esophagus cancer in men living in the Western** region during seven time periods of 1975-2009

| **Age** | | **Number of cancers in the time periods** | | | | | | |
| --- | --- | --- | --- | --- | --- | --- | --- | --- |
| Index, | Interval | 1975-79 | 1980-84 | 1985-89 | 1990-94 | 1995-99 | 2000-04 | 2005-09 |
| 1 | 20-24 | 1 | 0 | 0 | 0 | 1 | 1 | 0 |
| 2 | 25-29 | 0 | 0 | 1 | 0 | 4 | 2 | 2 |
| 3 | 30-34 | 3 | 4 | 3 | 6 | 7 | 10 | 3 |
| 4 | 35-39 | 8 | 2 | 8 | 15 | 8 | 12 | 14 |
| 5 | 40-44 | 10 | 13 | 33 | 31 | 39 | 27 | 29 |
| 6 | 45-49 | 40 | 40 | 45 | 60 | 81 | 95 | 74 |
| 7 | 50-54 | 75 | 63 | 71 | 95 | 110 | 166 | 174 |
| 8 | 55-59 | 104 | 120 | 144 | 151 | 163 | 214 | 260 |
| 9 | 60-64 | 147 | 156 | 187 | 187 | 216 | 219 | 314 |
| 10 | 65-69 | 163 | 167 | 200 | 235 | 244 | 241 | 263 |
| 11 | 70-74 | 121 | 138 | 157 | 232 | 236 | 231 | 212 |
| 12 | 75-79 | 60 | 80 | 116 | 146 | 163 | 192 | 219 |
| 13 | 80-84 | 39 | 43 | 61 | 71 | 99 | 148 | 153 |
| 14 | 85-89 | 23 | 16 | 34 | 45 | 49 | 56 | 57 |
| 15 | 90-94 | 5 | 8 | 6 | 2 | 17 | 25 | 18 |
| 16 | 95-99 | 2 | 1 | 0 | 1 | 5 | 1 | 2 |

**S1 Table 17. Distribution of the number of occurrences () of pancreatic cancer in women living in the Eastern** region during seven time periods of 1975-2009

| **Age** | | **Number of cancers in the time periods** | | | | | | |
| --- | --- | --- | --- | --- | --- | --- | --- | --- |
| Index, | Interval | 1975-79 | 1980-84 | 1985-89 | 1990-94 | 1995-99 | 2000-04 | 2005-09 |
| 1 | 20-24 | 3 | 2 | 2 | 7 | 2 | 3 | 2 |
| 2 | 25-29 | 4 | 5 | 3 | 2 | 4 | 3 | 3 |
| 3 | 30-34 | 0 | 11 | 6 | 5 | 3 | 8 | 15 |
| 4 | 35-39 | 16 | 16 | 19 | 20 | 25 | 22 | 23 |
| 5 | 40-44 | 16 | 28 | 30 | 34 | 44 | 46 | 54 |
| 6 | 45-49 | 50 | 57 | 64 | 62 | 85 | 114 | 136 |
| 7 | 50-54 | 120 | 127 | 90 | 109 | 128 | 171 | 211 |
| 8 | 55-59 | 200 | 198 | 185 | 167 | 189 | 215 | 296 |
| 9 | 60-64 | 225 | 279 | 281 | 243 | 247 | 243 | 347 |
| 10 | 65-69 | 282 | 322 | 401 | 346 | 370 | 343 | 374 |
| 11 | 70-74 | 275 | 336 | 379 | 434 | 457 | 442 | 400 |
| 12 | 75-79 | 244 | 288 | 360 | 385 | 441 | 476 | 487 |
| 13 | 80-84 | 159 | 222 | 237 | 248 | 293 | 322 | 387 |
| 14 | 85-89 | 93 | 100 | 116 | 124 | 146 | 154 | 208 |
| 15 | 90-94 | 21 | 27 | 32 | 34 | 43 | 56 | 70 |
| 16 | 95-99 | 3 | 6 | 11 | 6 | 12 | 4 | 13 |

**S1 Table 18. Distribution of the number of occurrences () of pancreatic cancer in women living in the Western** region during seven time periods of 1975-2009

| **Age** | | **Number of cancers in the time periods** | | | | | | |
| --- | --- | --- | --- | --- | --- | --- | --- | --- |
| Index, | Interval | 1975-79 | 1980-84 | 1985-89 | 1990-94 | 1995-99 | 2000-04 | 2005-09 |
| 1 | 20-24 | 3 | 3 | 0 | 1 | 3 | 0 | 0 |
| 2 | 25-29 | 5 | 1 | 4 | 1 | 7 | 3 | 5 |
| 3 | 30-34 | 10 | 8 | 10 | 11 | 13 | 12 | 18 |
| 4 | 35-39 | 17 | 24 | 34 | 32 | 30 | 40 | 32 |
| 5 | 40-44 | 41 | 44 | 59 | 56 | 72 | 88 | 71 |
| 6 | 45-49 | 105 | 94 | 89 | 84 | 145 | 160 | 172 |
| 7 | 50-54 | 181 | 180 | 152 | 171 | 228 | 278 | 295 |
| 8 | 55-59 | 279 | 304 | 243 | 242 | 264 | 358 | 416 |
| 9 | 60-64 | 366 | 362 | 386 | 292 | 333 | 360 | 495 |
| 10 | 65-69 | 351 | 378 | 406 | 450 | 367 | 375 | 487 |
| 11 | 70-74 | 286 | 346 | 379 | 421 | 386 | 405 | 397 |
| 12 | 75-79 | 218 | 239 | 248 | 302 | 316 | 332 | 371 |
| 13 | 80-84 | 115 | 133 | 168 | 145 | 186 | 234 | 275 |
| 14 | 85-89 | 49 | 58 | 68 | 50 | 68 | 80 | 115 |
| 15 | 90-94 | 9 | 10 | 18 | 13 | 17 | 17 | 28 |
| 16 | 95-99 | 1 | 1 | 5 | 2 | 1 | 1 | 5 |

**S1 Table 19. Distribution of the number of occurrences () of stomach cancer in women living in the Eastern** region during seven time periods of 1975-2009

| **Age** | | **Number of cancers in the time periods** | | | | | | |
| --- | --- | --- | --- | --- | --- | --- | --- | --- |
| Index, | Interval | 1975-79 | 1980-84 | 1985-89 | 1990-94 | 1995-99 | 2000-04 | 2005-09 |
| 1 | 20-24 | 3 | 2 | 3 | 0 | 0 | 8 | 5 |
| 2 | 25-29 | 3 | 1 | 10 | 10 | 7 | 4 | 9 |
| 3 | 30-34 | 12 | 12 | 11 | 18 | 20 | 15 | 14 |
| 4 | 35-39 | 17 | 20 | 21 | 30 | 24 | 33 | 36 |
| 5 | 40-44 | 36 | 29 | 31 | 30 | 46 | 55 | 57 |
| 6 | 45-49 | 47 | 54 | 47 | 47 | 56 | 73 | 88 |
| 7 | 50-54 | 91 | 66 | 56 | 63 | 80 | 91 | 114 |
| 8 | 55-59 | 158 | 115 | 90 | 93 | 84 | 111 | 107 |
| 9 | 60-64 | 167 | 153 | 150 | 129 | 116 | 107 | 128 |
| 10 | 65-69 | 230 | 206 | 196 | 180 | 138 | 124 | 143 |
| 11 | 70-74 | 221 | 257 | 246 | 248 | 220 | 183 | 185 |
| 12 | 75-79 | 272 | 246 | 272 | 299 | 248 | 223 | 192 |
| 13 | 80-84 | 195 | 233 | 269 | 244 | 250 | 246 | 210 |
| 14 | 85-89 | 128 | 160 | 161 | 139 | 175 | 167 | 146 |
| 15 | 90-94 | 38 | 55 | 71 | 68 | 73 | 80 | 73 |
| 16 | 95-99 | 8 | 11 | 13 | 16 | 25 | 20 | 16 |

**S1 Table 20. Distribution of the number of occurrences () of stomach cancer in women living in the Western** region during seven time periods of 1975-2009

| **Age** | | **Number of cancers in the time periods** | | | | | | |
| --- | --- | --- | --- | --- | --- | --- | --- | --- |
| Index, | Interval | 1975-79 | 1980-84 | 1985-89 | 1990-94 | 1995-99 | 2000-04 | 2005-09 |
| 1 | 20-24 | 3 | 2 | 2 | 0 | 6 | 4 | 3 |
| 2 | 25-29 | 6 | 6 | 14 | 8 | 8 | 9 | 13 |
| 3 | 30-34 | 17 | 16 | 17 | 8 | 20 | 26 | 21 |
| 4 | 35-39 | 18 | 36 | 27 | 30 | 33 | 35 | 35 |
| 5 | 40-44 | 39 | 33 | 42 | 53 | 62 | 40 | 65 |
| 6 | 45-49 | 59 | 44 | 64 | 46 | 60 | 75 | 85 |
| 7 | 50-54 | 79 | 82 | 80 | 71 | 69 | 99 | 115 |
| 8 | 55-59 | 131 | 111 | 101 | 97 | 94 | 118 | 145 |
| 9 | 60-64 | 137 | 146 | 147 | 136 | 125 | 127 | 121 |
| 10 | 65-69 | 170 | 184 | 184 | 200 | 183 | 161 | 132 |
| 11 | 70-74 | 191 | 200 | 189 | 205 | 214 | 196 | 174 |
| 12 | 75-79 | 203 | 224 | 240 | 224 | 231 | 238 | 201 |
| 13 | 80-84 | 195 | 184 | 161 | 195 | 225 | 219 | 235 |
| 14 | 85-89 | 129 | 123 | 122 | 138 | 139 | 159 | 131 |
| 15 | 90-94 | 32 | 50 | 47 | 61 | 59 | 61 | 59 |
| 16 | 95-99 | 1 | 5 | 13 | 14 | 16 | 18 | 16 |

**S1 Table 21. Distribution of the number of occurrences () of gallbladder cancer in women living in the Eastern** region during seven time periods of 1975-2009

| **Age** | | **Number of cancers in the time periods** | | | | | | |
| --- | --- | --- | --- | --- | --- | --- | --- | --- |
| Index, | Interval | 1975-79 | 1980-84 | 1985-89 | 1990-94 | 1995-99 | 2000-04 | 2005-09 |
| 1 | 20-24 | 0 | 0 | 0 | 0 | 0 | 0 | 1 |
| 2 | 25-29 | 1 | 0 | 1 | 0 | 0 | 1 | 1 |
| 3 | 30-34 | 0 | 1 | 0 | 0 | 1 | 2 | 4 |
| 4 | 35-39 | 1 | 1 | 1 | 3 | 1 | 3 | 4 |
| 5 | 40-44 | 6 | 5 | 6 | 6 | 6 | 14 | 12 |
| 6 | 45-49 | 10 | 6 | 6 | 8 | 14 | 21 | 16 |
| 7 | 50-54 | 20 | 17 | 24 | 18 | 18 | 20 | 22 |
| 8 | 55-59 | 32 | 32 | 34 | 30 | 26 | 30 | 35 |
| 9 | 60-64 | 51 | 55 | 50 | 56 | 31 | 38 | 48 |
| 10 | 65-69 | 80 | 72 | 75 | 65 | 57 | 47 | 50 |
| 11 | 70-74 | 66 | 100 | 80 | 87 | 67 | 61 | 62 |
| 12 | 75-79 | 96 | 106 | 89 | 97 | 75 | 74 | 60 |
| 13 | 80-84 | 85 | 82 | 82 | 74 | 69 | 67 | 59 |
| 14 | 85-89 | 60 | 75 | 48 | 51 | 45 | 40 | 43 |
| 15 | 90-94 | 18 | 23 | 24 | 14 | 16 | 17 | 9 |
| 16 | 95-99 | 2 | 3 | 5 | 5 | 4 | 6 | 1 |

**S1 Table 22. Distribution of the number of occurrences () of gallbladder cancer in women living in the Western** region during seven time periods of 1975-2009

| **Age** | | **Number of cancers in the time periods** | | | | | | |
| --- | --- | --- | --- | --- | --- | --- | --- | --- |
| Index, | Interval | 1975-79 | 1980-84 | 1985-89 | 1990-94 | 1995-99 | 2000-04 | 2005-09 |
| 1 | 20-24 | 0 | 0 | 0 | 0 | 0 | 0 | 0 |
| 2 | 25-29 | 1 | 0 | 0 | 2 | 0 | 0 | 0 |
| 3 | 30-34 | 1 | 1 | 1 | 1 | 1 | 2 | 1 |
| 4 | 35-39 | 1 | 2 | 5 | 8 | 1 | 4 | 7 |
| 5 | 40-44 | 8 | 6 | 6 | 9 | 4 | 4 | 10 |
| 6 | 45-49 | 12 | 7 | 14 | 8 | 12 | 16 | 13 |
| 7 | 50-54 | 24 | 22 | 17 | 27 | 18 | 25 | 24 |
| 8 | 55-59 | 26 | 37 | 28 | 23 | 29 | 34 | 34 |
| 9 | 60-64 | 31 | 46 | 41 | 37 | 32 | 34 | 41 |
| 10 | 65-69 | 59 | 56 | 59 | 46 | 47 | 42 | 50 |
| 11 | 70-74 | 67 | 58 | 58 | 72 | 61 | 52 | 62 |
| 12 | 75-79 | 69 | 77 | 62 | 63 | 55 | 68 | 44 |
| 13 | 80-84 | 53 | 61 | 53 | 47 | 35 | 55 | 67 |
| 14 | 85-89 | 51 | 41 | 35 | 36 | 28 | 31 | 34 |
| 15 | 90-94 | 5 | 8 | 15 | 10 | 12 | 13 | 18 |
| 16 | 95-99 | 2 | 4 | 2 | 5 | 2 | 2 | 1 |

**S1 Table 23. Distribution of the number of occurrences () of colon and rectum cancer in women living in the Eastern** region during seven time periods of 1975-2009

| **Age** | | **Number of cancers in the time periods** | | | | | | |
| --- | --- | --- | --- | --- | --- | --- | --- | --- |
| Index, | Interval | 1975-79 | 1980-84 | 1985-89 | 1990-94 | 1995-99 | 2000-04 | 2005-09 |
| 1 | 20-24 | 20 | 12 | 6 | 12 | 18 | 20 | 28 |
| 2 | 25-29 | 38 | 22 | 22 | 38 | 50 | 36 | 48 |
| 3 | 30-34 | 76 | 79 | 78 | 90 | 95 | 98 | 89 |
| 4 | 35-39 | 97 | 131 | 150 | 154 | 169 | 186 | 189 |
| 5 | 40-44 | 198 | 218 | 233 | 271 | 294 | 343 | 418 |
| 6 | 45-49 | 467 | 394 | 336 | 425 | 514 | 601 | 651 |
| 7 | 50-54 | 829 | 754 | 647 | 605 | 705 | 939 | 1131 |
| 8 | 55-59 | 1228 | 1204 | 1067 | 877 | 927 | 1128 | 1181 |
| 9 | 60-64 | 1549 | 1668 | 1649 | 1424 | 1231 | 1277 | 1197 |
| 10 | 65-69 | 1878 | 2061 | 2104 | 1938 | 1745 | 1568 | 1317 |
| 11 | 70-74 | 2034 | 2284 | 2288 | 2300 | 2260 | 1910 | 1523 |
| 12 | 75-79 | 1983 | 2283 | 2402 | 2431 | 2414 | 2315 | 1755 |
| 13 | 80-84 | 1608 | 1900 | 2101 | 2022 | 2191 | 2151 | 1795 |
| 14 | 85-89 | 861 | 1223 | 1261 | 1357 | 1437 | 1452 | 1290 |
| 15 | 90-94 | 249 | 390 | 500 | 506 | 630 | 583 | 549 |
| 16 | 95-99 | 50 | 60 | 105 | 102 | 115 | 115 | 96 |

**S1 Table 24. Distribution of the number of occurrences () of colon and rectum cancer in women living in the Western** region during seven time periods of 1975-2009

| **Age** | | **Number of cancers in the time periods** | | | | | | |
| --- | --- | --- | --- | --- | --- | --- | --- | --- |
| Index, | Interval | 1975-79 | 1980-84 | 1985-89 | 1990-94 | 1995-99 | 2000-04 | 2005-09 |
| 1 | 20-24 | 12 | 13 | 9 | 7 | 10 | 28 | 20 |
| 2 | 25-29 | 33 | 29 | 27 | 33 | 41 | 39 | 55 |
| 3 | 30-34 | 51 | 46 | 60 | 56 | 69 | 77 | 106 |
| 4 | 35-39 | 88 | 107 | 101 | 135 | 160 | 152 | 196 |
| 5 | 40-44 | 162 | 147 | 191 | 270 | 286 | 308 | 354 |
| 6 | 45-49 | 299 | 261 | 304 | 326 | 479 | 565 | 568 |
| 7 | 50-54 | 525 | 520 | 492 | 502 | 648 | 876 | 1053 |
| 8 | 55-59 | 772 | 850 | 771 | 650 | 798 | 927 | 1144 |
| 9 | 60-64 | 982 | 1051 | 1079 | 995 | 959 | 940 | 1133 |
| 10 | 65-69 | 1184 | 1314 | 1422 | 1314 | 1246 | 1201 | 1240 |
| 11 | 70-74 | 1263 | 1502 | 1624 | 1577 | 1644 | 1428 | 1262 |
| 12 | 75-79 | 1214 | 1451 | 1608 | 1589 | 1639 | 1668 | 1420 |
| 13 | 80-84 | 980 | 1102 | 1272 | 1385 | 1362 | 1489 | 1395 |
| 14 | 85-89 | 519 | 693 | 821 | 815 | 924 | 983 | 949 |
| 15 | 90-94 | 158 | 258 | 309 | 327 | 367 | 400 | 373 |
| 16 | 95-99 | 33 | 52 | 70 | 73 | 81 | 92 | 84 |

**S1 Table 25. Distribution of the number of occurrences () of liver cancer in women living in the Eastern** region during seven time periods of 1975-2009

| **Age** | | **Number of cancers in the time periods** | | | | | | |
| --- | --- | --- | --- | --- | --- | --- | --- | --- |
| Index, | Interval | 1975-79 | 1980-84 | 1985-89 | 1990-94 | 1995-99 | 2000-04 | 2005-09 |
| 1 | 20-24 | 1 | 4 | 3 | 3 | 3 | 1 | 4 |
| 2 | 25-29 | 4 | 8 | 1 | 6 | 1 | 7 | 4 |
| 3 | 30-34 | 3 | 2 | 3 | 5 | 4 | 8 | 8 |
| 4 | 35-39 | 6 | 4 | 9 | 2 | 10 | 11 | 9 |
| 5 | 40-44 | 5 | 10 | 8 | 6 | 12 | 15 | 12 |
| 6 | 45-49 | 11 | 6 | 13 | 16 | 18 | 28 | 40 |
| 7 | 50-54 | 18 | 25 | 17 | 14 | 37 | 48 | 62 |
| 8 | 55-59 | 25 | 35 | 27 | 23 | 30 | 55 | 102 |
| 9 | 60-64 | 41 | 41 | 38 | 48 | 45 | 54 | 56 |
| 10 | 65-69 | 29 | 41 | 51 | 52 | 73 | 61 | 72 |
| 11 | 70-74 | 51 | 44 | 57 | 65 | 76 | 79 | 66 |
| 12 | 75-79 | 31 | 50 | 48 | 61 | 78 | 71 | 80 |
| 13 | 80-84 | 26 | 23 | 39 | 50 | 55 | 63 | 78 |
| 14 | 85-89 | 8 | 13 | 15 | 17 | 29 | 30 | 30 |
| 15 | 90-94 | 2 | 4 | 4 | 7 | 3 | 5 | 16 |
| 16 | 95-99 | 0 | 0 | 1 | 0 | 2 | 1 | 4 |

**S1 Table 26. Distribution of the number of occurrences () of liver cancer in women living in the Western** region during seven time periods of 1975-2009

| **Age** | | **Number of cancers in the time periods** | | | | | | |
| --- | --- | --- | --- | --- | --- | --- | --- | --- |
| Index, | Interval | 1975-79 | 1980-84 | 1985-89 | 1990-94 | 1995-99 | 2000-04 | 2005-09 |
| 1 | 20-24 | 1 | 3 | 2 | 3 | 2 | 6 | 1 |
| 2 | 25-29 | 3 | 7 | 4 | 3 | 7 | 3 | 3 |
| 3 | 30-34 | 2 | 4 | 6 | 5 | 6 | 3 | 4 |
| 4 | 35-39 | 6 | 7 | 11 | 9 | 4 | 5 | 10 |
| 5 | 40-44 | 9 | 10 | 11 | 11 | 14 | 19 | 18 |
| 6 | 45-49 | 10 | 12 | 11 | 17 | 39 | 34 | 43 |
| 7 | 50-54 | 16 | 27 | 23 | 27 | 31 | 41 | 64 |
| 8 | 55-59 | 29 | 25 | 31 | 27 | 38 | 50 | 104 |
| 9 | 60-64 | 20 | 25 | 35 | 67 | 56 | 67 | 79 |
| 10 | 65-69 | 32 | 32 | 42 | 40 | 74 | 89 | 83 |
| 11 | 70-74 | 32 | 36 | 45 | 60 | 80 | 100 | 115 |
| 12 | 75-79 | 42 | 44 | 38 | 68 | 70 | 85 | 103 |
| 13 | 80-84 | 23 | 23 | 30 | 28 | 41 | 57 | 66 |
| 14 | 85-89 | 7 | 18 | 16 | 15 | 21 | 27 | 38 |
| 15 | 90-94 | 0 | 2 | 3 | 4 | 4 | 8 | 13 |
| 16 | 95-99 | 0 | 1 | 0 | 0 | 1 | 0 | 1 |

**S1 Table 27. Distribution of the number of occurrences () of esophagus cancer in women living in the Eastern** region during seven time periods of 1975-2009

| **Age** | | **Number of cancers in the time periods** | | | | | | |
| --- | --- | --- | --- | --- | --- | --- | --- | --- |
| Index, | Interval | 1975-79 | 1980-84 | 1985-89 | 1990-94 | 1995-99 | 2000-04 | 2005-09 |
| 1 | 20-24 | 0 | 0 | 0 | 0 | 0 | 1 | 0 |
| 2 | 25-29 | 0 | 1 | 1 | 2 | 0 | 0 | 0 |
| 3 | 30-34 | 0 | 0 | 0 | 2 | 2 | 2 | 1 |
| 4 | 35-39 | 4 | 4 | 4 | 1 | 7 | 5 | 2 |
| 5 | 40-44 | 14 | 8 | 9 | 9 | 9 | 15 | 11 |
| 6 | 45-49 | 30 | 29 | 15 | 26 | 18 | 38 | 23 |
| 7 | 50-54 | 42 | 52 | 35 | 37 | 37 | 40 | 61 |
| 8 | 55-59 | 77 | 76 | 72 | 67 | 51 | 64 | 53 |
| 9 | 60-64 | 72 | 96 | 85 | 75 | 64 | 66 | 74 |
| 10 | 65-69 | 78 | 85 | 101 | 86 | 88 | 64 | 77 |
| 11 | 70-74 | 63 | 70 | 90 | 89 | 113 | 91 | 84 |
| 12 | 75-79 | 46 | 64 | 64 | 86 | 90 | 97 | 94 |
| 13 | 80-84 | 31 | 36 | 66 | 55 | 67 | 76 | 81 |
| 14 | 85-89 | 26 | 27 | 31 | 42 | 45 | 75 | 50 |
| 15 | 90-94 | 8 | 12 | 14 | 17 | 18 | 21 | 21 |
| 16 | 95-99 | 0 | 1 | 4 | 1 | 4 | 6 | 6 |

**S1 Table 28. Distribution of the number of occurrences () of esophagus cancer in women living in the Western** region during seven time periods of 1975-2009

| **Age** | | **Number of cancers in the time periods** | | | | | | |
| --- | --- | --- | --- | --- | --- | --- | --- | --- |
| Index, | Interval | 1975-79 | 1980-84 | 1985-89 | 1990-94 | 1995-99 | 2000-04 | 2005-09 |
| 1 | 20-24 | 0 | 0 | 0 | 0 | 0 | 1 | 0 |
| 2 | 25-29 | 1 | 0 | 0 | 1 | 1 | 0 | 1 |
| 3 | 30-34 | 0 | 0 | 1 | 1 | 1 | 1 | 0 |
| 4 | 35-39 | 1 | 2 | 1 | 0 | 2 | 1 | 3 |
| 5 | 40-44 | 6 | 2 | 1 | 7 | 3 | 8 | 5 |
| 6 | 45-49 | 14 | 1 | 10 | 6 | 9 | 23 | 19 |
| 7 | 50-54 | 29 | 23 | 15 | 21 | 17 | 28 | 34 |
| 8 | 55-59 | 61 | 42 | 41 | 31 | 34 | 36 | 51 |
| 9 | 60-64 | 50 | 56 | 70 | 61 | 40 | 40 | 41 |
| 10 | 65-69 | 72 | 59 | 75 | 80 | 67 | 57 | 73 |
| 11 | 70-74 | 40 | 53 | 72 | 71 | 76 | 76 | 58 |
| 12 | 75-79 | 29 | 56 | 78 | 61 | 78 | 63 | 69 |
| 13 | 80-84 | 22 | 39 | 44 | 48 | 62 | 68 | 56 |
| 14 | 85-89 | 15 | 20 | 19 | 24 | 30 | 36 | 44 |
| 15 | 90-94 | 7 | 6 | 11 | 18 | 14 | 23 | 14 |
| 16 | 95-99 | 1 | 1 | 4 | 1 | 2 | 4 | 5 |
